# A Combined Model Based on Bone Mineral Density for Noninvasive Prediction of Prognosis in Non‐Small Cell Lung Cancer Patients Receiving Immune Checkpoint Inhibitors: A Multicenter Retrospective Study

**DOI:** 10.1002/mco2.70398

**Published:** 2025-09-21

**Authors:** Bingxin Gong, Yusheng Guo, Qi Wan, Jie Lou, Yi Li, Tingjie Xiong, Peng Mo, Yiqun Chen, Xiaowen Liu, Zilong Wu, Zhaokai Wang, Dongxuan Wei, Xi Zhang, Hongxiang Zeng, Xiaofei Zhang, Hui Wang, Lian Yang

**Affiliations:** ^1^ Department of Radiology Union Hospital Tongji Medical College Huazhong University of Science and Technology Wuhan China; ^2^ Hubei Provincial Clinical Research Center For Precision Radiology & Interventional Medicine Wuhan China; ^3^ Hubei Key Laboratory of Molecular Imaging Wuhan China; ^4^ Department of Radiology the Key Laboratory of Advanced Interdisciplinary Studies Center National Center for Respiratory Medicine the First Affiliated Hospital of Guangzhou Medical University Guangzhou China; ^5^ Department of Radiotherapy 900th Hospital of Joint Logistics Support Force Fujian Medical University Fuzhou China; ^6^ Department of Respiratory and Critical Care Medicine Affiliated Hospital of Nantong University Medical School of Nantong University Nantong China; ^7^ Department of Radiology Shenzhen People's Hospital (The Second Clinical Medical College of Jinan University The First Affiliated Hospital of Southern University of Science and Technology) Shenzhen China; ^8^ Cancer Center Union Hospital Tongji Medical College Huazhong University of Science and Technology Wuhan China; ^9^ Department of Thoracic Surgery Union Hospital Tongji Medical College Huazhong University of Science and Technology Wuhan China; ^10^ Department of Orthopedics Union Hospital Tongji Medical College Huazhong University of Science and Technology Wuhan China; ^11^ CT Business Unit Neusoft Medical Systems Co. Ltd Shenyang China; ^12^ Intelligent Imaging Software R&D Division Neusoft Medical Systems Co. Ltd Shenyang China; ^13^ Center For Translational Medicine Union Hospital Tongji Medical College Huazhong University of Science and Technology Wuhan China; ^14^ Department of Medical Genetics School of Basic Medicine Tongji Medical College Huazhong University of Science and Technology Wuhan China

**Keywords:** bone mineral density, bone mineral density decrease, immunotherapy, immune checkpoint inhibitors, non‐small cell lung cancer, osteoporosis

## Abstract

The prognostic value of baseline bone mineral density (BMD) and posttreatment BMD decrease (BMDD) in non‐small cell lung cancer (NSCLC) patients receiving immune checkpoint inhibitor (ICI) treatment remains unclear. We assembled data of 2096 patients with advanced NSCLC from five institutions to develop a combined model incorporating BMD/BMDD and clinical characteristics for noninvasive prognosis prediction. BMD was automatically assessed using a deep learning‐based method. Compared with the physiological BMD group and the non‐severe BMDD group, the pathological BMD group and the severe BMDD group had shorter progression‐free survival (PFS) (hazard ratio [HR]: 1.19, *p* = 0.003; and HR: 1.19, *p* = 0.002, respectively) and overall survival (OS) (HR: 1.31, *p* < 0.001; and HR: 1.30, *p* < 0.001). Compared with the single BMD/BMDD model, the combined model had higher Harrell's concordance indexes (*c*‐indexes) (PFS: 0.580 and OS: 0.654). Transcriptomic analysis of 130 patients from the NSCLC radiogenomic cohort revealed upregulation of epithelial–mesenchymal transition, inflammatory, and hypoxia pathways, and increased macrophage infiltration in tumors of patients with pathological BMD. This study showed that lower baseline BMD and more severe BMDD are associated with poorer prognosis. BMD in combination with clinical characteristics can help to improve risk stratification and prognosis prediction.

## Graphical abstract

In this study, we obtained the bone mineral density (BMD) values of each patient before and after immune checkpoint inhibitor (ICI) treatment by an automatic algorithm based on deep learning, and calculated the extent of BMD decrease (BMDD) and age‐adjusted standard BMD (BMDA). Our study found that BMD decreased after ICI treatment, and lower pretreatment BMD and more severe posttreatment BMDD have been shown to be associated with shorter progression‐free survival (PFS) and overall survival (OS). Further transcriptomic analyses suggest that patients with low BMD have a more aggressive molecular signature of tumors.

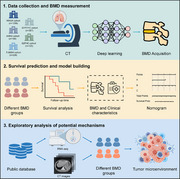

## 1 Introduction

Treatment of non‐small cell lung cancer (NSCLC) has progressed significantly in recent years. Immunotherapy based on immune checkpoint inhibitors (ICIs) has significantly improved the survival rate of patients with advanced NSCLC [1]. However, disease control and long‐term survival rates remain dismal. Only 10%–20% of patients with advanced NSCLC show durable responses to treatment, and the 5‐year overall survival (OS) rate still does not exceed 30% [2]. Considering that not all patients can benefit from ICI treatment, there is an urgent need to find reliable and efficient biomarkers that can help patients avoid unnecessary immune‐related adverse events (irAEs) and reduce the financial burden.

In recent years, substantial research works have been committed to identify potential biomarkers to predict the efficacy of ICIs. Although molecular markers such as programmed cell death‐ligand 1 (PD‐L1) expression and tumor mutational burden (TMB) show promise, their clinical application remains limited due to challenges such as tissue sampling, invasiveness, and high costs [3, 4]. Meanwhile, a growing body of research has explored the regulatory role of the tumor immune microenvironment in antitumor immunity, such as immune cell infiltration and microbiome modulation [5, 6, 7].

In this context, imaging biomarkers are attracting attention as noninvasive and easily accessible tools for predicting the efficacy of immunotherapy. Body composition parameters derived from routine computed tomography (CT) scans, such as skeletal muscle mass and adipose tissue distribution, could reflect nutritional status, metabolic reserve, and systemic inflammation of patients with cancer [8]. Several studies have demonstrated that body composition parameters are closely associated with the clinical outcomes of NSCLC patients receiving immunotherapy [9, 10].

In addition to sarcopenia and weight loss, osteoporosis is also a common and significant concern for patients with malignant tumors [11]. Osteopenia is defined as a condition of abnormally low bone mineral density (BMD), with *T*‐score measured by dual‐energy x‐ray absorptiometry (DXA) as the gold standard [12]. It was reported that the trabecular attenuation value of the first lumbar (L1) vertebra measured noninvasively by CT imaging was significantly correlated with the *T*‐score of DXA and can be used as an imaging surrogate parameter [13]. BMD was considered to play a predictive role in several serious diseases, including cancer, and has received increasing attention as an emerging prognostic biomarker [14]. A previous study has shown that lower baseline BMD is associated with poor prognosis in NSCLC patients with brain metastases (BM) [15]. There is increasing evidence for a link between bone metabolism and immune regulation [16]. Cytokines secreted by tumor cells could stimulate osteoclasts and subsequently activate the NF‐κB (RANK)/RANK ligand (RANKL) pathway, which is related to the effectiveness of checkpoint blockade in cancer treatment [17, 18]. Additionally, bone loss was a continuous pathological process under the long‐lasting negative effects of the tumor. Therefore, besides the baseline BMD, the extent of decline in BMD during treatment and follow‐up should also be investigated with great care.

It is worth noting that no study has reported the extent of the decrease in BMD in patients with NSCLC treated with ICIs and its association with prognosis. This multicenter study aims to construct independent and composite models using patients' baseline BMD, BMD decrease (BMDD) during follow‐up, and related clinical characteristics to explore their feasibility in predicting the efficacy and prognosis of immunotherapy in NSCLC patients in order to provide a noninvasive tool for treatment decision‐making and prognosis assessment of NSCLC. Additionally, transcriptomic analysis of radiogenomics datasets was performed to explore the potential biological mechanisms of BMD prediction.

## 2 Results

### 2.1 Patient Characteristics

To explore the predictive value and potential mechanism of BMD in the efficacy of immunotherapy, this study included 2226 patients to establish an imaging‐prognostic cohort and an imaging‐gene‐prognosis cohort (2096 and 130 patients, respectively), all of whom had high‐quality CT images, clinical and prognostic data. The imaging‐prognostic cohort consisted of 2096 patients with NSCLC treated with ICIs who were retrospectively recruited from five academic centers between April 2017 and January 2024 (Wuhan Union Hospital [WHUH] cohort, 1256 patients; Affiliated Hospital of Nantong University [AHNU], 100 patients; Fujian 900th Hospital of Joint Logistics Support Force [FJ900H] cohort, 228 patients; The First Affiliated Hospital of Guangzhou Medical University [GYFYY] cohort, 125 patients; and Shenzhen People's Hospital [SZPH] cohort, 387 patients). In this cohort, we divided patients into pathological BMD group (BMD < age‐adjusted standard BMD [BMDA]) and physiological BMD group (BMD ≥ BMDA) according to the relationship between BMD and BMDA, and divided patients into severe BMDD group (BMDD < −12.9) and non‐severe BMDD group (BMDD ≥ −12.9) according to the optimal BMDD cutoff value obtained by X‐tile software (Figure S1). Table 1 shows the clinical and pathological characteristics of patients in different groups of this cohort. All patients had advanced (Stage III or IV) NSCLC and received ICI treatment in their first anticancer treatment. Specific ICI drugs are shown in Table S1. The pathological BMD group had higher proportion of females than the physiological BMD group (18.2% vs. 14.6%, *p* = 0.039); however, this did not affect the predictive power because physiological BMD and pathological BMD were divided based on BMDA, which already took age and gender into account. Meanwhile, the proportions of patients with baseline bone metastasis were different between the severe BMDD group and the non‐severe BMDD group (19.7% vs. 23.5%, *p* = 0.042). Other than this, there were no significant differences in baseline characteristics among the groups. In addition, we constructed an imaging‐gene‐prognosis cohort consisting of 130 primary NSCLC patients from the public database, and matched the transcriptome information to explore the biological basis of BMD prediction.

**TABLE 1:** Baseline characteristics of patients in different BMD groups.

Characteristics	Pathological BMD group (*n* = 1391)	Physiological BMD group (*n* = 705)	*p* value	Severe BMDD group (*n* = 822)	Non‐severe BMDD group (*n* = 1274)	*p* value
Gender			0.039			0.687
Male	1138 (81.8)	602 (85.4)		679 (82.6)	1061 (83.3)	
Female	253 (18.2)	103 (14.6)		143 (17.4)	213 (16.7)	
Age (year)^a^	64 (58, 69)	63 (56, 68)	0.057	64 (58, 68)	63 (57, 69)	0.833
Pathological types			0.313			0.082
Adenocarcinoma	668 (48.0)	348 (49.4)		380 (46.2)	636 (49.9)	
Squamous cell carcinoma	595 (42.8)	281 (39.9)		368 (44.8)	508 (39.9)	
Other^b^	128 (9.2)	76 (10.8)		74 (9.0)	130 (10.2)	
Stages			0.606			0.495
Stage III	478 (34.7)	251 (35.9)		294 (36)	435 (34.5)	
Stage IV	899 (65.3)	449 (64.1)		523 (64)	825 (65.5)	
BMI (kg/m^2^)^a^	22.4 (20.2, 24.4)	22.3 (20.4, 24.4)	0.932	22.5 (20.4, 24.5)	22.3 (20.2, 24.3)	0.126
Diabetes	134 (9.6)	77 (10.9)	0.354	82 (10)	129 (10.1)	0.911
Hypertension	335 (24.1)	172 (24.4)	0.888	205 (25)	302 (23.7)	0.516
Smoking	722 (57.1)	374 (57.6)	0.832	433 (56.6)	663 (57.8)	0.618
Drinking	350 (29.1)	169 (27)	0.361	213 (28.9)	306 (28)	0.661
Hyperlipidemia	340 (25.1)	155 (22.3)	0.150	208 (25.7)	287 (23.2)	0.197
Total bilirubin (µmol/L)^a^	9.4 (7.4, 12.7)	9.6 (7.4, 12.9)	0.397	9.5 (7.4, 12.9)	9.4 (7.4, 12.6)	0.548
Albumin (g/L)^a^	38.5 (35.5, 41.4)	39 (35.4, 41.7)	0.218	38.9 (35.5, 41.7)	38.6 (35.4, 41.3)	0.301
Calcium (mmol/L)^a^	2.2 (2.1, 2.3)	2.2 (2.1, 2.3)	0.981	2.2 (2.1, 2.3)	2.2 (2.1, 2.3)	0.336
NLR^a^	3.2 (2.1, 5.3)	3.4 (2.1, 5.7)	0.078	3.2 (2.1, 5.5)	3.3 (2.1, 5.4)	0.649
PLR^a^	179.5 (127.8, 258.0)	179.3 (121.6, 267.0)	0.894	182.6 (125.4, 264.1)	176.3 (126.3, 258.9)	0.889
PD‐L1 expression			0.114			0.577
TPS < 1%	338 (66.9)	154 (61.1)		194 (66.2)	298 (64.2)	
TPS ≥ 1%	167 (33.1)	98 (38.9)		99 (33.8)	166 (35.8)	
ECOG status			0.503			0.758
0	508 (36.5)	268 (38.0)		301 (36.6)	475 (37.3)	
≥ 1	883 (63.5)	437 (62.0)		521 (63.4)	799 (62.7)	
Bone metastasis	312 (22.4)	149 (21.1)	0.499	162 (19.7%)	299 (23.5%)	0.042
Brain metastases	201 (14.5)	86 (12.2)	0.157	113 (13.7)	174 (13.7)	0.954
Corticosteroid use	506 (36.4)	244 (34.6)	0.425	292 (35.5)	458 (35.9)	0.842
Immunotherapy regimen			0.802			0.155
ICI	177 (12.7)	618 (87.7)		93 (11.3)	171 (13.4)	
ICI with chemotherapy	1214 (87.3)	87 (12.3)		729 (88.7)	1103 (86.6)	

*Note*: Data are the number of patients with percentages in parentheses unless otherwise noted.

Abbreviations: BMD, bone mineral density; BMDD, bone mineral density decrease; BMI, body mass index; ECOG, Eastern Cooperative Oncology Group; ICI, immune checkpoint inhibitor; IQR, interquartile range; NLR, neutrophil‐to‐lymphocyte ratio; PD‐L1, programmed cell death‐ligand 1; PLR, platelet count‐to‐lymphocyte ratio; TPS, tumor proportion score.

^a^ Data are medians, with IQRs in parentheses.

^b^ Non‐small cell lung cancer other than squamous cell carcinoma and adenocarcinoma.

### 2.2 Analysis of BMD

Whether in the training set or the test set, the segmentation results of muscle, fat, and spine areas have a high dice similarity coefficient (DSC) (Table S2). Additionally, we randomly selected 500 adult patients who underwent DXA examinations from the medical records system. The DXA *T*‐scores of the L1 vertebra and the BMD calculated by the automated method were obtained. The correlation scatter plot demonstrated a moderate association between the automated BMD measurements and corresponding DXA *T*‐scores (*R* = 0.617, *p* < 0.001; Figure S2A). Furthermore, significant differences in L1 vertebral BMD were observed among the osteoporosis, osteopenia, and normal cohorts (all *p* < 0.001; Figure S2B). We analyzed the BMD results of 2096 patients before and after treatment and found that after four cycles of the ICI treatment, the BMD value showed a significant decrease (136 vs. 124, *p* < 0.001; Figure S3).

### 2.3 Tumor Response

The tumor responses of each group are shown in Table S3. Overall, there was no significant difference in the short‐term treatment effect between the groups. The disease control rate (DCR) and objective response rate (ORR) in the pathological BMD group were 87.3% and 33.4%, respectively, and in the physiological BMD group were 88.5% and 33.9%, respectively (DCR, *p* = 0.443; ORR, *p* = 0.829). Similarly, there was no statistical difference in DCR (87.3% vs. 88.0%, *p* = 0.661) and ORR (33.9% vs. 33.4%, *p* = 0.783) between the severe BMDD group and the non‐severe BMDD group. The alluvial plot shows the proportional relationship between different groups and specific tumor responses (Figure S4).

### 2.4 Survival Analysis and Subgroup Analysis

**FIGURE 1:**
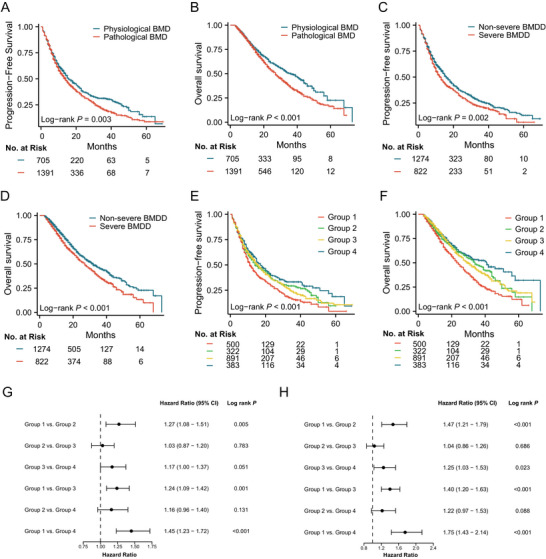
Kaplan–Meier curves of PFS and OS in the independent and combined groups. (A–D) Kaplan–Meier curve of PFS and OS in physiological BMD, pathological BMD, non‐severe BMDD, and severe BMDD groups. Kaplan–Meier curves of PFS (E) and OS (F) for the BMD and BMDD combined groups, and forest plots (G, H) showed the hazard ratio ​​and log‐rank *p* values ​​between different combined groups. Group 1 represents the combination of pathological BMD and severe BMDD, Group 2 represents the combination of physiological BMD and severe BMDD, Group 3 represents the combination of pathological BMD and non‐severe BMDD, and Group 4 represents the combination of physiological BMD and non‐severe BMDD. BMD, bone mineral density; BMDD, bone mineral density decrease; OS, overall survival; PFS, progression‐free survival.

As of the final follow‐up deadline (August 2024), the median follow‐up time was 16.0 months (interquartile range [IQR]: 9.6, 29.0). Specifically, 899 of 1391 patients in the pathological BMD group (64.6%) and 431 of 705 patients in the physiological BMD group (61.1%) experienced disease progression, of whom 645 (46.4%) and 296 (42.0%) patients died, respectively. Compared with the physiological BMD group, the progression‐free survival (PFS) (median 14.0 months [95% CI: 13.0, 15.0] vs. 16.0 months [95% CI: 15.0, 19.0]; hazard ratios [HRs]: 1.19 [95% CI: 1.06, 1.34]; *p* = 0.003; Figure 1A) and OS (median 27.0 months [95% CI: 25.0, 29.0] vs. 36.0 months [95% CI: 32.0, 42.0]; HR: 1.31 [95% CI: 1.14, 1.51]; *p* < 0.001; Figure 1B) of the pathological BMD group were significantly shorter. Similarly, disease progression occurred in 582 (582/822, 70.8%) and 748 (748/1274, 58.7%) patients in the severe BMDD and non‐severe BMDD groups, respectively, including 447 (447/822, 54.4%) and 494 (494/1274, 38.8%) deaths. The PFS (median 12.5 months [95% confidence intervals (95% CI): 11.0, 14.0] vs. 16.0 months [95% CI: 15.0, 18.0]; HR 1.19 [95% CI: 1.06, 1.32]; *p* = 0.002; Figure 1C) and OS (median 27.0 months [95% CI: 24.0, 29.2] vs. 32.0 months [95% CI: 29.0, 36.0]; HR 1.30 [95% CI: 1.14, 1.48]; *p* < 0.001; Figure 1D) of the severe BMDD group were significantly shorter than those of the non‐severe BMDD group. To demonstrate the robustness of the results, we performed validation in each independent cohort (Figure S5A–T). The results from different cohorts were similar to or trended in the same direction as the primary outcome, especially in terms of stratified OS. Meanwhile, we compared the survival rates of patients in each group at four time points (1, 2, 3, and 5 years) and found that compared to the physiological BMD group and the non‐severe BMD group, the survival rates were significantly shorter in the pathological BMD group and the severe BMD group, and the majority of them could be shown to be statistically different (Table S4). Specifically, patients with pathological BMD showed significantly lower survival rates at Years 2 and 3 compared to those with physiological BMD. Meanwhile, patients with severe BMDD exhibited significantly lower survival rates at all time points assessed than those with non‐severe BMDD. This suggests that the severity of BMDD may have a sustained and significant impact on the efficacy of immunotherapy. To further explore the predictive power of BMD and BMDD, we permuted and combined each group to form new groups. As expected, the same‐direction combination of BMD and BMDD (pathological BMD and severe BMDD group, and physiological BMD and non‐severe BMDD group) had the highest stratification power, whether it was stratified PFS or OS. And the non‐same‐direction combination of BMD and BMDD (physiological BMD and severe BMDD group, and pathological BMD and non‐severe BMDD group) had a general stratification power, and the results were marginally positive (Figure 1E–H). Furthermore, the relationship of clinical characteristics and survival outcomes of patients in different BMD and BMDD groups is shown in heat maps (Figure S6).

To further investigate the possible factors affecting prognosis and adjust for related confounding variables, we performed COX regression analysis (Table 2; Table S5). In the univariate regression analysis, pathological BMD, severe BMDD, Stage IV, lower albumin, higher neutrophil‐to‐lymphocyte ratio (NLR), higher platelet count‐to‐lymphocyte ratio (PLR), lower PD‐L1 expression, higher Eastern Cooperative Oncology Group (ECOG) status, bone metastasis, BM, corticosteroid use, and ICI monotherapy were simultaneously identified as potential risk factors for shorter PFS or/and OS, and these covariates were included in the multivariate regression analysis. In multivariate analysis, pathological BMD (HR: 1.20, 95% CI: 1.06, 1.35; *p* = 0.003), severe BMDD (HR: 1.24, 95% CI: 1.11, 1.39; *p* < 0.001), Stage IV (HR: 1.27, 95% CI: 1.11, 1.45; *p* < 0.001), lower albumin (HR: 1.02, 95% CI: 1.01, 1.03; *p* < 0.001), higher PLR (HR: 1.00, 95% CI: 1.00, 1.00; *p* = 0.038), higher ECOG status (HR: 1.38, 95% CI: 1.22, 1.56; *p* < 0.001), bone metastasis (HR: 1.30, 95% CI: 1.14, 1.49; *p* < 0.001), and corticosteroid use (HR: 1.13, 95% CI: 1.01, 1.27; *p* = 0.034) were identified as being associated with shorter PFS; pathological BMD (HR: 1.70, 95% CI: 1.32, 2.19; *p* < 0.001), severe BMDD (HR: 1.54, 95% CI: 1.23, 1.93; *p* < 0.001), Stage IV (HR: 1.50, 95% CI: 1.14, 1.96; *p* = 0.004), lower albumin (HR: 1.05, 95% CI: 1.03, 1.08; *p* < 0.001), lower PD‐L1 expression (HR: 1.30, 95% CI: 1.02, 1.64; *p* = 0.034), higher ECOG status (HR: 1.53, 95% CI: 1.15, 2.05; *p* = 0.003), and corticosteroid use (HR: 1.35, 95% CI: 1.08, 1.70; *p* = 0.010) were independent predictors of death. Furthermore, we found that KRAS (HR: 0.50, 95% CI: 0.28, 0.89; *p* = 0.019) and MET (HR: 0.59, 95% CI: 0.31, 1.12; *p* = 0.106) gene mutations emerged as potential predictive factors for longer PFS. In multivariate analysis, KRAS mutation retained only marginal significance (HR: 0.55, 95% CI: 0.30, 1.00; *p* = 0.052) (Table S6). We did not find the association between gene mutations and OS (Table S7).

**TABLE 2:** Univariate and multivariate Cox proportional hazards analyses for OS.

Parameter	Univariate analysis		Multivariate analysis	
	Hazard ratio (95% CI)	*p* value	Hazard ratio (95% CI)	*p* value
Groups (BMD)				
Physiological BMD	Reference		Reference	
Pathological BMD	1.31 (1.14, 1.51)	< 0.001	1.70 (1.32, 2.19)	< 0.001
Groups (BMDD)				
Non‐severe BMDD	Reference			
Severe BMDD	1.30 (1.15, 1.48)	< 0.001	1.54 (1.23, 1.93)	< 0.001
Gender				
Male	Reference			
Female	1.12 (0.95, 1.33)	0.162		
Age	1.00 (1.00, 1.00)	0.685		
Pathological types				
Adenocarcinoma	Reference			
Squamous cell carcinoma	1.01 (0.88, 1.15)	0.936	1.19 (0.92, 1.52)	0.186
Other^a^	1.31 (1.07, 1.61)	0.010	1.34 (0.93, 1.92)	0.117
Stages				
Stage III	Reference		Reference	
Stage IV	1.23 (1.07, 1.41)	0.004	1.50 (1.14, 1.96)	0.004
BMI (kg/m^2^)	1.01 (0.99, 1.03)	0.467		
Diabetes				
No	Reference			
Yes	1.04 (0.84, 1.29)	0.725		
Hypertension				
No	Reference			
Yes	0.96 (0.82, 1.11)	0.564		
Smoking				
No	Reference			
Yes	0.97 (0.85, 1.10)	0.630		
Drinking				
No	Reference			
Yes	0.99 (0.85, 1.15)	0.898		
Hyperlipidemia				
No	Reference			
Yes	0.89 (0.76, 1.05)	0.171		
Total bilirubin (µmol/L)	1.00 (0.99, 1.01)	0.761		
Albumin (g/L)	0.97 (0.95, 0.98)	< 0.001	0.95 (0.93, 0.97)	< 0.001
Calcium (mmol/L)	0.88 (0.57, 1.35)	0.548		
NLR	1.01 (1.00, 1.02)	0.057	1.00 (0.98, 1.03)	0.954
PLR	1.00 (1.00, 1.00)	< 0.001	1.00 (1.00, 1.00)	0.834
PD‐L1 expression				
TPS ≥ 1%	Reference		Reference	
TPS < 1%	1.22 (0.97, 1.53)	0.091	1.30 (1.02, 1.64)	0.034
ECOG status				
0	Reference		Reference	
≥1	1.68 (1.45, 1.95)	< 0.001	1.53 (1.15, 2.05)	0.003
Bone metastasis				
No	Reference		Reference	
Yes	1.21 (1.04, 1.41)	0.013	1.15 (0.88, 1.50)	0.308
Brain metastases				
No	Reference			
Yes	0.99 (0.82, 1.19)	0.881		
Corticosteroid use				
No	Reference		Reference	
Yes	1.31 (1.15, 1.49)	< 0.001	1.35 (1.08, 1.70)	0.010
Immunotherapy regimen				
ICI	Reference			
ICI with chemotherapy	0.93 (0.78, 1.11)	0.438		

*Note*: All hazard ratios are shown for one‐unit increments for each variable unless otherwise indicated.

Abbreviations: BMD, bone mineral density; BMDD, bone mineral density decrease; BMI, body mass index; CI, confidence interval; ECOG, Eastern Cooperative Oncology Group; ICI, immune checkpoint inhibitor; NLR, neutrophil‐to‐lymphocyte ratio; OS, overall survival; PD‐L1, programmed cell death‐ligand 1; PLR, platelet count‐to‐lymphocyte ratio; TPS, tumor proportion score.

^a^ Non‐small cell lung cancer other than squamous cell carcinoma and adenocarcinoma.

The effects of BMD and BMDD on PFS and OS were consistently observed in subgroups based on baseline characteristics (Figures S7–S10). Except for the female, other pathological types, diabetes, and hypertension subgroups, the pathological BMD and severe BMDD groups had higher risks of shortened PFS and OS than the physiological BMD and non‐severe BMDD groups in the remaining subgroups. Specifically, female patients were only susceptible to the impact of BMD loss on OS (HR: 1.45, 95% CI: 1.08, 1.95), hypertensive patients were only susceptible to the impact of baseline BMD on OS (HR: 1.57, 95% CI: 1.16, 2.15), and other pathological types and diabetic patients were not sensitive to BMD and BMD loss, whether in PFS or OS.

### 2.5 Prognostic Models

**FIGURE 2:**
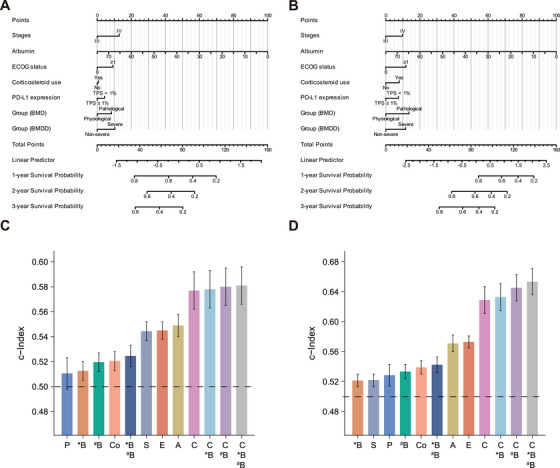
Combined nomograms for survival and *c*‐indexes for integration of combinations of clinical‐imaging features. (A) Progression‐free survival nomogram. (B) Overall survival nomogram. (C) The *c*‐index of progression‐free survival. (D) The *c*‐index of overall survival. A, albumin; ^*^B, bone mineral density; ^#^B, bone mineral density decrease; BMD, bone mineral density. BMDD, bone mineral density decrease; C, Clinical (including clinical stages, albumin, ECOG status, corticosteroid use, and PD‐L1 expression); Co, corticosteroid use; E, ECOG status; ECOG, Eastern Cooperative Oncology Group; P, PD‐L1 expression; PD‐L1, programmed cell death‐ligand 1; S, clinical stages; TPS, tumor proportion score.

We developed two prognostic nomograms to predict PFS (Figure 2A) and OS (Figure 2B) at 1, 2, and 3 years in patients. The variables in the nomogram included stages, albumin, ECOG status, corticosteroid use, PD‐L1 expression, BMD, and BMDD, which were determined based on multivariate COX regression for OS. The Harrell's concordance index was used to evaluate the predictive performance of the prognostic nomogram, and the *c*‐indexes of the PFS and OS nomograms were 0.580 (95% Cl: 0.565, 0.595) and 0.654 (95% Cl: 0.636, 0.671), respectively. Considering that clinical variables, BMD, and BMDD had independent predictive values, we combined these clinical imaging features to form the new prognostic models and calculated the *c*‐indexes of different prognostic models (Figure 2C,D). The results showed that all models had predictive ability (the Harrell's concordance indexes [*c*‐indexes] > 0.5), and the model combining all variables had the highest consistency index, whether predicting PFS or OS. At the same time, we performed a correlation analysis on the above clinical imaging features. The results showed that the correlation coefficients between the parameters were low (Figure S11; Table S8), indicating that these clinical imaging features can be used as complementary information for predicting prognosis.

### 2.6 NSCLC Radiogenomics Cohort Analyses Results

**FIGURE 3:**
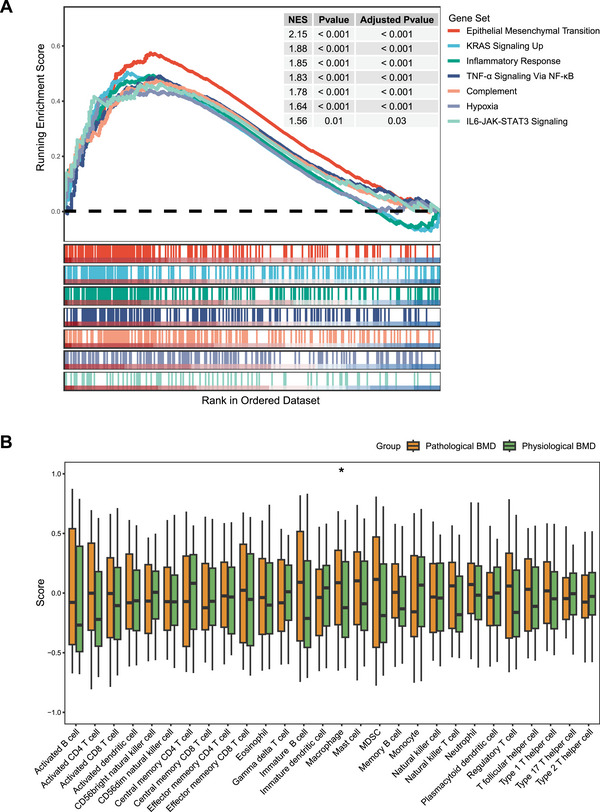
(A) GSEA analysis of Hallmark gene sets comparing differences between pathological and physiological BMD. (B) The differences in tumor immune infiltration by immune deconvolution between pathological and physiological BMD. NES, normalized enrichment score; BMD, bone mineral density. *, *p* < 0.05.

The NSCLC radiogenomics cohort (*n* = 130) was predominantly male (73.8%) with a median age of 69 years (IQR: 63.8, 76.0). Patients were divided into the pathological BMD group (*n* = 73) and the physiological BMD (*n* = 57) group based on BMDA. The physiological BMD group had a longer OS than the pathological BMD group, although there was no statistical difference (HR: 1.44 [95% Cl: 0.79, 2.64]; *p* = 0.235) (Figure S5U). Gene set enrichment analysis (GSEA) revealed that tumors from patients with pathological BMD have increased expression of epithelial–mesenchymal transition, inflammatory (inflammatory response, tumor necrosis factor‐α [TNF‐α], complement, and interleukin‐6 [IL‐6]), and hypoxia (Figure 3A). In addition, compared with the physiologic BMD group, tumors from patients with pathological BMD demonstrated an increased degree of macrophage infiltration (*p* = 0.043), but the differences between the remaining immune cells were not statistically significant (Figure 3B).

## 3 Discussion

Based on a multicenter cohort, this study explored the relationship between BMD‐related indicators (including baseline BMD and BMDD) and long‐term prognosis in NSCLC patients receiving ICI treatment. Moreover, given the impact of age and gender on BMD, we introduced BMDA adjusted for the age and gender of each patient. Our results showed that BMD decreased after ICI treatment, and lower baseline BMD and more severe BMDD were associated with shorter PFS and OS. Subsequently, we further explored other clinical characteristics that affected patient prognosis and constructed the combined model based on BMD/BMDD and clinical characteristics, which performed well in prognostic stratification.

Although DXA is the gold standard for assessing BMD, in patients with severe vertebral degeneration or vertebral fracture, the DXA method is susceptible to the influence of degenerative structures, fractures, and so forth, leading to inaccurate measurements [19]. An increasing number of studies have shown that CT scans are suitable for predicting vertebral fractures and serially measuring bone loss and correlate well with BMD measured by DXA [13, 20]. Similarly, we verified this result in an independent DXA cohort. In addition, chest CT is a routine examination item for NSCLC diagnosis, staging, efficacy evaluation, and follow‐up. Measuring BMD using chest CT does not require additional costs, time, scanning equipment, and radiation exposure, and therefore is more likely to be popularized in clinical practice. The membrane‐free quantitative computed tomography (QCT) is a complementary form of BMD measurement, but traditional QCT requires a body phantom and specialized analysis software. The present study established a deep learning pipeline to automatically extract the BMD of the L1 vertebra from the patient's CT images using a membrane‐free QCT analysis method. At the same time, an attention mechanism was added to the deep learning pipeline, which improved the segmentation ability of muscle, fat, and vertebrae by simulating the human ability to selectively focus on key parts when processing information, and calibrated BMD through the CT values ​​of the psoas major muscle, subcutaneous fat, and all vertebrae to obtain more accurate BMD results.

Previous studies have demonstrated the unique and important value of BMD in assessing the prognosis of patients with tumors. Ilic et al. [15]. evaluated preoperative BMD in patients with NSCLC‐related BM undergoing surgery and found that lower preoperative BMD was associated with worse median OS and higher 1‐year mortality. Similar conclusions can be extended to other tumors. For example, several studies have found that lower BMD is an independent risk factor for poor prognosis in patients with hepatocellular carcinoma (HCC) and gastrointestinal tumors [21, 22]. Our previous study explored the predictive value of baseline BMD in NSCLC patients receiving ICIs [23]. However, considering that bone loss in cancer patients may be a long‐term process, especially under the dual burden of tumor and immunotherapy, baseline BMD alone is difficult to fully reflect the overall picture of BMD during tumor treatment and its relationship with tumor and tumor treatment. Therefore, this study evaluated the changes in BMD during immunotherapy and analyzed its relationship with immunotherapy efficacy and prognosis. Meanwhile, considering that BMD is easily affected by gender and age, it is not rigorous enough to simply divide baseline BMD into high and low groups in previous studies. Therefore, the present study introduced BMDA adjusted for age and gender, and divided patients into a physiological BMD group and a pathological BMD group based on the relationship between baseline BMD and BMDA. Furthermore, to enhance the robustness of the data and the generalizability of the results, this study incorporated multicenter data. We found that BMD and BMDD generally demonstrated consistent prognostic stratification ability across the five cohorts. However, in some cohorts, their stratification performance was only marginally significant or even did not reach statistical significance. These discrepancies may be related to the population heterogeneity among the cohorts. Additionally, variations in sample size may also have influenced the manifestation of statistical effects.

In this study, we observed varying degrees of BMD reduction in NSCLC patients treated with ICIs. Notably, the pathogenesis of BMD reduction is multifactorial. For patients with tumors, tumor cells release a variety of cytokines such as interleukin‐1 (IL‐1), IL‐6, and TNF‐α, which can stimulate osteoclast differentiation via multiple mechanisms and disrupt the balance of bone remodeling [24, 25]. In the middle and late stages of the disease, tumor cells could alter metabolic processes through various pathways, leading to cancer cachexia syndrome and bone loss [26]. In addition, recent studies have shown that patients treated with ICIs can develop systemic bone loss or localized bone lesions, which are unlikely to be attributed to tumor‐induced osteolysis, suggesting that programmed cell death 1 (PD‐1) blockade may have an impact on the bone remodeling process [27, 28]. A preclinical study found that PD‐1 deficiency or nivolumab treatment could inhibit osteoclastogenesis and rescue the low bone volume phenotype induced after inoculation with Lewis Lung carcinoma cells [29]. To elucidate the underlying mechanisms, several studies have investigated the role of PD‐1/PD‐L1 expression in the skeletal microenvironment, immune cell subsets, as well as hypoxia and the tumor microenvironment in tumor‐induced bone disease [30, 31].

The field of osteoimmunology, which aims to explore the complex interactions between the skeletal and immune systems, has been attracting increasing attention [32]. Although previous studies have found that BMD may be associated with clinical outcomes in cancer patients receiving immunotherapy, the underlying mechanisms remain unclear. In our exploratory analyses, we found that the TNF‐α signaling via NF‐kB and IL6/JAK/STAT3 signaling pathways was activated in tumors with pathological BMD. In fact, several studies have shown that TNF‐α and IL‐6 can contribute to bone loss by promoting osteoclast formation through the RANKL/RANK axis [33]. RANK/RANKL signaling can promote the generation of regulatory T cells (Tregs) and increase production of cytokines, thereby diminishing the efficacy of immune checkpoint blockade [17, 34]. IL‐6, in particular, has been reported to impair anti‐PD‐L1 efficacy by limiting the antitumor function of cytotoxic T cells [35, 36]. In addition, tumors with pathological BMD exhibit activation of epithelial–mesenchymal transition (EMT) and hypoxic pathways and macrophage infiltration. It has been shown that EMT can lead to an immunosuppressive tumor microenvironment by generating or attracting immunosuppressive cells such as tumor‐associated macrophages and myeloid‐derived suppressor cells [37]. At the same time, hypoxia induces macrophage polarization and accumulation of regulatory T cells (Treg) to form an immunosuppressive microenvironment [38]. The upregulation of these pathways may create an immunosuppressive microenvironment, which may reduce patients’ response to ICIs. Overall, our transcriptomic data provide preliminary molecular evidence for a potential link between bone metabolism disorders and the efficacy of immunotherapy. Further animal experiments and prospective studies are needed to elucidate the direct impact of bone‐immune cross‐regulation on immunotherapy outcomes.

Integrating our research findings and the underlying mechanistic basis, incorporating baseline BMD and monitoring BMDD during treatment into the clinical management of immunotherapy holds promise for assisting clinicians in early identification of high‐risk patients and providing a basis for individualized therapy decisions. Specifically, for patients with pretreatment BMD < BMDA, clinicians should conduct a comprehensive assessment incorporating other clinical indicators (such as tumor stage, albumin levels) to optimize the treatment plan, and consider the use of immunomodulators and bone‐protective agents (e.g., anti‐RANKL drugs) to improve bone metabolism and hopefully enhance the response to immunotherapy. Concurrently, dynamic monitoring of BMD levels throughout the treatment course is essential. For patients with severe BMDD, clinicians should re‐evaluate based on their tumor treatment response: for responders, continuation of combination therapy with bone protection and intensified follow‐up may be considered; for non‐responders, adjusting the treatment strategy to explore a more suitable comprehensive treatment plan should be considered. Future studies are still needed to further validate the clinical benefits of this integrated management strategy.

Our study has several limitations. First, we only analyzed the value of BMD in NSCLC patients receiving ICI treatment and combination regimens. However, chemotherapy alone and targeted therapy are still important treatments for NSCLC patients. Therefore, more samples are needed in the future to explore the value of BMD in other treatments. Second, we note that the relatively low *c*‐indexes of the combined model predicting PFS and OS may limit the clinical utility of our model. In future studies, we plan to incorporate more comprehensive body composition biomarkers or integrate multi‐omics data to enhance the predictive power of our model. Third, our biological interpretability analysis is primarily based on sequencing data from early‐stage patients in public databases, whereas our study focuses on advanced NSCLC patients. Although similar studies have previously employed this research paradigm [39, 40, 41], future population‐specific biological validation remains necessary. Fourth, due to the inconsistent timing of CT scan follow‐ups for each patient during the observation period, we did not obtain BMD measurements at multiple time points. Future prospective studies are necessary to assess BMD at multiple standardized time points during follow‐up in order to further evaluate the prognostic value of BMD and BMDD. Finally, this is a retrospective study, and bias and confounding are inevitable, although we have compensated for this problem by employing appropriate study design and statistical methods. While our study is the largest multicenter cohort study reported to date, prospective studies with large samples and long follow‐up are still needed to validate our results.

## 4 Conclusion

In conclusion, this multi‐cohort study revealed that lower BMD and more severe BMDD are markers of poor prognosis in NSCLC patients treated with ICIs. Further transcriptomic analyses suggest that patients with low BMD have a more aggressive molecular signature of tumors. Therefore, our study advocates that assessment of bone status before and during immunotherapy may provide crucial information to guide treatment decisions.

## 5 Material and Methods

### 5.1 Study Design and Patient Selection

**FIGURE 4:**
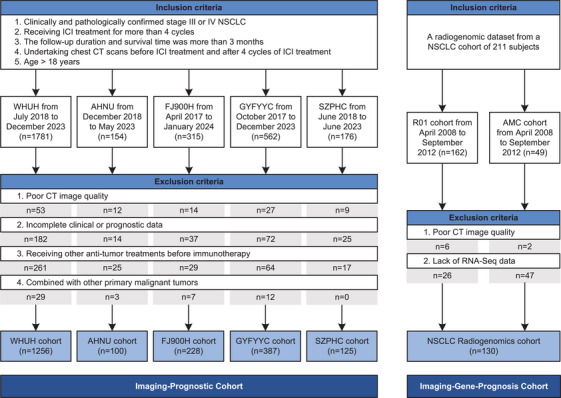
Flow diagrams show the patient recruitment process. The left picture includes five medical centers, and the cohorts on the right are from the public database. ICI, immune checkpoint inhibitor; NSCLC, non‐small cell lung cancer.

Retrospective collection of data on patients with NSCLC treated with ICIs from April 2017 to January 2024 at five academic institutions, including WHUH, AHNU, FJ900H, GYFYY, and SZPH, with consistent inclusion and exclusion criteria. Inclusion criteria were as follows: (1) patients who were diagnosed with advanced NSCLC according to the radiologic and histological or cytological examination, (2) patients treated with ICIs for at least four consecutive cycles, (3) the follow‐up duration and survival time was more than 3 months, (4) performing CT examination before and after ICI treatment, and (5) age > 18 years. Exclusion criteria were as follows: (1) patients with missing CT images or with artifacts that interfere with analysis, (2) patients with incomplete clinical information or prognostic data, (3) patients who received other antitumor treatments before immunotherapy, and (4) patients with a combination of a primary malignant tumor other than lung cancer. Meanwhile, a radiogenomics dataset was collected from an NSCLC cohort, including the R01 cohort and the AMC cohort, in which patients underwent surgical treatment [42]. Further screening was performed according to the following exclusion criteria: (1) poor CT image quality; (2) lack of RNA‐sequencing (RNA‐Seq) data; and finally, 130 subjects were included in the NSCLC radiogenomics cohort in our study. The detailed patient inclusion process is shown in Figure 4.

Clinical and pathological data were collected from the initial admission and discharge medical records of patients, including patient demographics, biochemical data, treatment information, tumor‐related information, pathology, and CT examination data. Hypertension, diabetes, and hyperlipidemia included previous diagnosis and confirmed diagnosis by the first examination after admission. Smoking was defined as smoking more than one cigarette a day for at least 1 year. Tumor staging was conducted according to the ninth edition of TNM Classification for lung cancer [43].

### 5.2 Assessment of BMD

**FIGURE 5:**
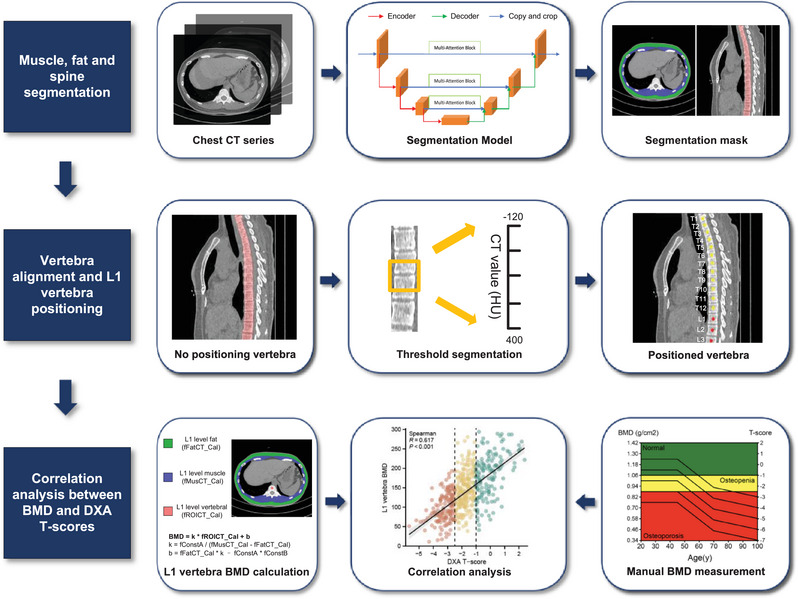
Automated BMD measurement workflow. BMD, bone mineral density; CT, computed tomography; DXA, dual‐energy x‐ray absorptiometry; L1, first lumbar.

All CT imaging data were obtained from five institutions (WHUH cohort, AHNU cohort, FJ900H cohort, GYFYY cohort, and SZPH cohort). Specific CT scanners and parameters for each institution were presented in Table S9. BMD was defined as the trabecular attenuation value of the L1 vertebra and was automatically measured using the QCT BMD analysis method (Figure 5). Firstly, 200 patients were randomly selected from the total cohort and divided into training, test, and validation sets at a ratio of 7:2:1 to train the segmentation model (Figure S12). The specific parameters of model training were shown in Table S10. The image annotation of the training set was completed by a radiologist with 7 years of osteomyology imaging experience using ITK‐snap software (version 3.8, http://www.itksnap.org). To ensure the accuracy of the annotation results, all the annotated images were checked and modified one by one by the team members. The segmentation model used Residual U‐Net (ResUnet) as the basic framework and added an attention mechanism to automatically segment the muscle, fat, and spine areas efficiently and accurately. Second, when the model completed the segmentation, the spine was marked from top to bottom, the L1 vertebra was automatically located, the center point of the L1 cone was calculated, and the region of interest (ROI) was divided according to the center point. The average CT value of the vertebral ROI at the L1 level was calculated and recorded as *fROICT_Cal*; the average CT value of the muscle at the L1 level was calculated and recorded as *fMusCT_Cal*; the average CT value of the fat at the L1 level was calculated and recorded as *fFatCT_Cal*. Finally, the BMD of the L1 vertebra after calibration was calculated by the calibration equation ($BMD=k\times fROICT_{Cal}+b$; $k=\frac{fConstA}{\mathrm{fMusCT}\_\mathrm{Cal}\;-\;\mathrm{fFatCT}\_\mathrm{Cal}}$; b=fFatCT_Cal×k−fConstA×fConstB; *fConstA* and *fConstB* represent the correction factors). The calculation process provided by Neusoft Medical Advanced Visualization Workspace 4.0 Bone Density. To verify the reliability of the automatic method, we randomly selected 500 adult patients who underwent DXA examinations from the medical records system to establish a DXA cohort. We recorded the DXA *T*‐scores of the L1 vertebra for each patient while simultaneously using the automated method to obtain the BMD. Both the automated method and DXA *T*‐score recording were performed blinded to clinical information. All completed images were reviewed by a second radiologist with 26 years of experience in osteomyology imaging to avoid interference with BMD assessment due to bone metastasis, fracture, bone destruction, focal sclerosis, osteolytic lesions, and so forth (the images of such patients were excluded due to poor image quality). Any disagreement was resolved through group discussion. The extent of BMDD was defined as the difference in BMD before ICI treatment and after four cycles of treatment divided by the baseline BMD, and it was calculated using the formula: BMDD = [(baseline BMD − posttreatment BMD)/baseline BMD] ×100%. The BMDA was introduced [44], which used the following formulae: BMDA for men = 308.82 − 2.49 × Age in years; BMDA for women = 311.84 − 2.41 × Age in years. This formula could estimate standard BMD in each sex using data on ages. Patients with baseline BMD < BMDA were defined as the pathological BMD group, and patients with baseline BMD ≥ BMDA were defined as the physiological BMD group. The optimal cutoff value of BMDD was obtained by using X‐tile software to divide the patients into the severe BMDD group and the non‐severe BMDD group.

### 5.3 Follow‐Up and End Points

All patients were followed until August 2024. Response and progression were assessed by follow‐up CT or MRI compared with pretreatment imaging based on the Response Evaluation Criteria in Solid Tumors (RECIST), version 1.1 [45]. PFS was defined as the duration from the initial ICI treatment until the occurrence of tumor progression or death of the patient. OS was defined as the period from the commencement of ICI treatment to the last follow‐up or the occurrence of patient mortality. Tumor response was judged based on clinical and imaging data 1 month after treatment, which was presented in the form of complete response (CR), partial response (PR), stable disease (SD), and progressive disease (PD). DCR was calculated as the proportion of patients with CR and PR plus SD, and ORR was calculated as the proportion of patients with CR plus PR.

### 5.4 Transcriptomic Analyses of NSCLC Radiogenomics Cohort

We included a cohort of 130 patients with available RNA‐seq data and performed an exploratory analysis of gene expression patterns according to BMD [42]. CT images were downloaded, and BMD was measured using the same approach described above [46]. Differential expression analyses were performed using the R package “DESeq2” [47]. GSEA was used to evaluate differences in the Molecular Signatures Database (MSigDB) hallmark gene set collection [48, 49]. Based on the previously published set of immune cell genes, we evaluated the infiltration of immune cells in 130 samples using single‐sample gene enrichment analysis (ssGSEA) [50].

### 5.5 Statistical Analysis

The paired or independent Student's *t*‐test was used to compare the continuous variables, and the chi‐square test was used to calculate the discrete variables. Correlations between BMDD/BMD and clinical data were performed using point‐biserial correlation analysis and phi correlation analysis. The DSC was used to evaluate the accuracy of automated segmentation results, and the correlation between the L1 vertebra BMD and DXA *T*‐scores was assessed using Spearman's correlation coefficient. X‐tile software (Yale University, version 3.6.1) was used to determine the optimal cutoff values of BMDD. All parameters with a *p* value < 0.10 in the univariate analysis were entered into a multivariate Cox regression model, and HR and 95% CI were calculated. The prognostic models of BMD‐related, clinical‐related, and their combinations were established, and the performance of models was assessed using the *c*‐indexes. Kaplan–Meier survival analysis was used to compare the survival differences among patients with different scores in each model. We also conducted exploratory subgroup analyses based on covariates of clinical interest, and HRs with 95% CIs were reported within each subgroup. Immune cell types with significant differences were identified by the Wilcoxon test. All the tests conducted were two‐tailed, and a *p* value < 0.05 indicated statistical significance. The data were analyzed using SPSS software, version 26.0 (IBM, Chicago, IL, USA), and R software, version 4.3.0 (R Foundation), by G.B.X., L.J., and G.Y.S.

## Author Contributions

Conception and design: B.G., Y.G., and L.Y. Administrative support: L.Y., X.Z., and H.W. Collection of data: Y.L., T.X., P.M., Y.C., Q.W., and X.L. Data analysis and interpretation: B.G., J.L., Z.W., Z.W., and D.W. Algorithm support: X.Z. and H.Z. Manuscript writing: B.G., Y.G., and Q.W. Responsible for the overall content: L.Y. All authors have read and approved the final manuscript.

## Ethics Statement

This retrospective study was approved by the Ethics Committee of Tongji Medical College, Huazhong University of Science and Technology (No. S048), Affiliated Hospital of Nantong University (No. 2018‐K020), Fujian 900th Hospital of Joint Logistics Support Force (2023‐097), The First Affiliated Hospital of Guangzhou Medical University (ES‐2024‐K173‐01), and Shenzhen People's Hospital (LL‐KY‐2021058).

## Consent

The requirement for informed consent was waived.

## Conflicts of Interest

Authors Xi Zhang and Hongxiang Zeng are employees of Neusoft Medical Systems Co. Ltd but have no potential relevant financial or nonfinancial interests to disclose. The other authors declare no conflicts of interest.

## Supporting information




**Table S1**: ICI drugs used by patients in different BMD groups.**Table S2**: DSC of the segmentation results of muscle, fat, and spine areas in the training and test sets.**Table S3**: Tumor Response in different BMD groups.**Table S4**: Survival rate in different BMD groups at different time periods.**Table S5**: Univariate and multivariate Cox proportional hazards analyses for PFS.**Table S6**: Univariate and multivariate Cox proportional hazards analyses for PFS with EGFR, ALK, KRAS and MET.**Table S7**: Univariate and multivariate Cox proportional hazards analyses for OS with EGFR, ALK, KRAS and MET.**Table S8**: Correlation coefficient between the individual parameters.**Table S9**: CT scanners and parameters of the five institutions.**Table S10**: Segmentation model training parameters.**Figure S1**: The optimal cutoff value based on OS for BMDD classification was determined by the X‐tile software. **(A)** X‐tile plots. **(B)** BMDD frequency histogram. OS, overall survival; BMDD, bone mineral density decrease.**Figure S2 (A)**: Scatter plot showing the correlation between L1 vertebral BMD and corresponding DXA T‐scores. Red dots represent osteoporosis cohorts (T‐score ≤ ‐2.5); yellow dots represent osteopenia cohorts (‐2.5 < T‐score < ‐1); green dots represent normal cohorts (T‐score ≥ ‐1). **(B)** Box plot demonstrating the differences in L1 vertebral BMD among cohorts with osteoporosis, osteopenia, and normal. *** indicates *p* < 0.001. L1, first lumbar; BMD, bone mineral density; DXA, dual‐energy X‐ray absorptiometry.**Figure S3**: Bar graph shows the decrease in BMD before (first BMD) and after (second BMD) treatment. BMD, bone mineral density.**Figure S4**: Alluvial plot of the correspondence between patients categorized according to baseline BMD, immunotherapy response, and BMDD. BMD, bone mineral density; BMDD, bone mineral density decrease; PD, progressive disease; PR, partial response; SD, stable disease.**Figure S5**: Kaplan‐Meier curves of PFS and OS in different BMD and BMDD groups from five institutions and public database (NSCLC radiogenomics cohort). **(A‐D)** WHUH cohort. **(E‐H)** FJ900H cohort. **(I‐L)** GYFYY cohort. **(M‐P)** AHNU cohort. **(Q‐T)** SZPH cohort. **(U)** NSCLC radiogenomics cohort. PFS, progression‐free survival; OS, overall survival; BMD, bone mineral density; BMDD, bone mineral density decrease; NSCLC, non‐small cell lung cancer; WHUH, Wuhan Union Hospital; FJ900H, Fujian 900th Hospital of Joint Logistics Support Force; GYFYY, The First Affiliated Hospital of Guangzhou Medical University; AHNU, Affiliated Hospital of Nantong University; SZPH, Shenzhen People's Hospital.**Figure S6**: Heat maps show the difference of clinical features and survival outcomes in patients in different BMD **(A)** and BMDD **(B)** groups. BMD, bone mineral density; OS, overall survival; BMI, Body Mass index; BMDD, bone mineral density decrease. ^*^Non‐small cell lung cancer other than squamous cell carcinoma and adenocarcinoma.**Figure S7**: Subgroup analyses of progression‐free survival between the physiological BMD and pathological BMD groups. Hazard ratios were derived from univariate cox model for each subgroup. Dashed line indicates Hazard ratio of 1. BMD, bone mineral density; CI, confidence interval. ^*^Non‐small cell lung cancer other than squamous cell carcinoma and adenocarcinoma.**Figure S8**: Subgroup analyses of overall survival between the physiological BMD and pathological BMD groups. Hazard ratios were derived from univariate cox model for each subgroup. Dashed line indicates Hazard ratio of 1. BMD, bone mineral density; CI, confidence interval. ^*^Non‐small cell lung cancer other than squamous cell carcinoma and adenocarcinoma.**Figure S9**: Subgroup analyses of progression‐free survival between the non‐severe BMDD and severe BMDD groups. Hazard ratios were derived from univariate cox model for each subgroup. Dashed line indicates Hazard ratio of 1. BMDD, bone mineral density decrease; CI, confidence interval. ^*^Non‐small cell lung cancer other than squamous cell carcinoma and adenocarcinoma.**Figure S10**: Subgroup analyses of overall survival between the non‐severe BMDD and severe BMDD groups. Hazard ratios were derived from univariate cox model for each subgroup. Dashed line indicates Hazard ratio of 1. BMDD, bone mineral density decrease; CI, confidence interval. ^*^Non‐small cell lung cancer other than squamous cell carcinoma and adenocarcinoma.**Figure S11**: Heat map of correlation coefficients between the individual parameters. A, albumin; S, clinical stages; E, ECOG status; Co, corticosteroid use; P, PD‐L1 expression; ^*^B, bone mineral density; ^#^B, bone mineral density decrease.**Figure S12**: Segmentation model training flow chart.

## Data Availability

All study data analyzed in this study can be provided upon reasonable request to the corresponding author.
