# Supplementary material for: A Combined Model Based on Bone Mineral Density for Noninvasive Prediction of Prognosis in Non‐Small Cell Lung Cancer Patients Receiving Immune Checkpoint Inhibitors: A Multicenter Retrospective Study
Source: MedComm (2020). 2025 Sep 21;6(10):e70398. doi: 10.1002/mco2.70398 (PMC12450769; doi:10.1002/mco2.70398)

**SUPPLEMENTAL MATERIAL**

**A combined model based on bone mineral density for non-invasive prediction of prognosis in non-small cell lung cancer patients receiving immune checkpoint inhibitors: a multicenter retrospective study**

Bingxin Gong^1,2,3#^, Yusheng Guo^1,2,3#^, Qi Wan^4#^, Jie Lou^1,2,3^, Yi Li^1,2,3^, Tingjie Xiong^5^, Peng Mo^5^, Yiqun Chen^6^, Xiaowen Liu^7^, Zilong Wu^8^, Zhaokai Wang^9^, Dongxuan Wei^10^, Xi Zhang^11^, Hongxiang Zeng^12^, Xiaofei Zhang^13*^, Hui Wang^14*^, Lian Yang^1,2,3*^

^1^Department of Radiology, Union Hospital, Tongji Medical College, Huazhong University of Science and Technology, Wuhan, China.

^2^Hubei Provincial Clinical Research Center for Precision Radiology & Interventional Medicine, Wuhan 430022, China.

^3^Hubei Key Laboratory of Molecular Imaging, Wuhan, China.

^4^Department of Radiology, the Key Laboratory of Advanced Interdisciplinary Studies Center, National Center for Respiratory Medicine, the First Affiliated Hospital of Guangzhou Medical University, Guangzhou, China.

^5^Department of Radiotherapy, 900th Hospital of Joint Logistics Support Force, Fujian Medical University, Fuzhou, China.

^6^Department of Respiratory and Critical Care Medicine, Affiliated Hospital of Nantong University, Medical School of Nantong University, Nantong, China.

^7^Department of Radiology, Shenzhen People’s Hospital (The Second Clinical Medical College of Jinan University, The First Affiliated Hospital of Southern University of Science and Technology), Shenzhen, Guangdong, China.

^8^Cancer Center, Union Hospital, Tongji Medical College, Huazhong University of Science and Technology, Wuhan, China.

^9^Department of Thoracic Surgery, Union Hospital, Tongji Medical College, Huazhong University of Science and Technology, Wuhan, China.

^10^Department of Orthopedics, Union Hospital, Tongji Medical College, Huazhong University of Science and Technology, Wuhan, China.

^11^CT Business Unit, Neusoft Medical Systems Co. Ltd, Shenyang, China.

^12^Intelligent Imaging Software R&D Division, Neusoft Medical Systems Co. Ltd, Shenyang, China.

^13^Center for Translational Medicine, Union Hospital, Tongji Medical College, Huazhong University of Science and Technology, Wuhan, China.

^14^Department of Medical Genetics, School of Basic Medicine, Tongji Medical College, Huazhong University of Science and Technology, Wuhan, China.

^#^Bingxin Gong, Yusheng Guo and Qi Wan contributed equally.

^*^Correspondence:

Lian Yang, Department of Radiology, Union Hospital, Tongji Medical College, Huazhong University of Science and Technology, No.1277 Jiefang Avenue, Wuhan, China; E-mail: yanglian@hust.edu.cn;

Hui Wang, Department of Medical Genetics, School of Basic Medicine, Tongji Medical College, Huazhong University of Science and Technology, Wuhan, China; E-mail: wanghuipitt@hust.edu.cn;

Xiaofei Zhang, Center for Translational Medicine, Union Hospital, Tongji Medical College, Huazhong University of Science and Technology, Wuhan, China; E-mail: zhangxf17@hust.edu.cn.

**Table of Contents: page**

**Supplemental Tables**

• **Table S1** ICI drugs used by patients in different BMD groups.………………..............................................................................5

• **Table S2** DSC of the segmentation results of muscle, fat, and spine areas in the training and test sets........……………….........6

• **Table S3** Tumor Response in different BMD groups…………………………………………………..…………………………7

• **Table S4** Survival rate in different BMD groups at different time periods……………………………………………………….8

• **Table S5** Univariate and multivariate Cox proportional hazards analyses for PFS…………………………………………....9-10

• **Table S6** Univariate and multivariate Cox proportional hazards analyses for PFS with EGFR, ALK, KRAS and MET…...…...11

• **Table S7** Univariate and multivariate Cox proportional hazards analyses for OS with EGFR, ALK, KRAS and MET….....…..12

• **Table S8** Correlation coefficient between the individual parameters……………………………………………………….……13

• **Table S9** CT scanners and parameters of the five institutions………………………..…………………..………………………14

• **Table S10** Segmentation model training parameters………………………………………………….....……………………….15

**Supplemental Figures and Figure Legends**

• **Figure S1** The optimal cutoff value based on OS for BMDD classification was determined by the X-tile software. **(A)** X-tile plots. **(B)** BMDD frequency histogram. OS, overall survival; BMDD, bone mineral density decrease……………………………16

• **Figure S2** **(A)** Scatter plot showing the correlation between L1 vertebral BMD and corresponding DXA T-scores. Red dots represent osteoporosis cohorts (T-score ≤ -2.5); yellow dots represent osteopenia cohorts (-2.5 < T-score < -1); green dots represent normal cohorts (T-score ≥ -1). **(B)** Box plot demonstrating the differences in L1 vertebral BMD among cohorts with osteoporosis, osteopenia, and normal. *** indicates *P* < 0.001. L1, first lumbar; BMD, bone mineral density; DXA, dual-energy X-ray absorptiometry.………………………………………………………………….………………………………….…………17

• **Figure S3** Bar graph shows the decrease in BMD before (first BMD) and after (second BMD) treatment. BMD, bone mineral density.…………………………………………………………………………………………………...……………….…………18

• **Figure S4** Alluvial plot of the correspondence between patients categorized according to baseline BMD, immunotherapy response, and BMDD. BMD, bone mineral density; BMDD, bone mineral density decrease; PD, progressive disease; PR, partial response; SD, stable disease…………………………………………………………………………………...…………………….19

• **Figure S5** Kaplan-Meier curves of PFS and OS in different BMD and BMDD groups from five institutions and public database (NSCLC Radiogenomics cohort). **(A-D)** WHUH cohort. **(E-H)** FJ900H cohort. **(I-L)** GYFYY cohort. **(M-P)** AHNU cohort. **(Q-T)** SZPH cohort. **(U)** NSCLC Radiogenomics cohort. PFS, progression-free survival; OS, overall survival; BMD, bone mineral density; BMDD, bone mineral density decrease; NSCLC, non-small cell lung cancer; WHUH, Wuhan Union Hospital; FJ900H, Fujian 900th Hospital of Joint Logistics Support Force; GYFYY, The First Affiliated Hospital of Guangzhou Medical University; AHNU, Affiliated Hospital of Nantong University; SZPH, Shenzhen People's Hospital…………………………………....….20-21

• **Figure S6** Heat maps show the difference of clinical features and survival outcomes in patients in different BMD **(A)** and BMDD **(B)** groups. BMD, bone mineral density; OS, overall survival; BMI, Body Mass index; BMDD, bone mineral density decrease. ^*^Non-small cell lung cancer other than squamous cell carcinoma and adenocarcinoma…………………………………………….22

• **Figure S7** Subgroup analyses of progression-free survival between the physiological BMD and pathological BMD groups. Hazard ratios were derived from univariate cox model for each subgroup. Dashed line indicates Hazard ratio of 1. BMD,
bone mineral density; CI, confidence interval. ^*^Non-small cell lung cancer other than squamous cell carcinoma and adenocarcinoma..…………………………………………………………………………………………………………………....23

• **Figure S8** Subgroup analyses of overall survival between the physiological BMD and pathological BMD groups. Hazard ratios were derived from univariate cox model for each subgroup. Dashed line indicates Hazard ratio of 1. BMD, bone mineral density; CI, confidence interval. ^*^Non-small cell lung cancer other than squamous cell carcinoma and adenocarcinoma...………………..24

• **Figure S9** Subgroup analyses of progression-free survival between the non-severe BMDD and severe BMDD groups. Hazard ratios were derived from univariate cox model for each subgroup. Dashed line indicates Hazard ratio of 1. BMDD, bone mineral density decrease; CI, confidence interval. ^*^Non-small cell lung cancer other than squamous cell carcinoma and adenocarcinoma……………………………………………………………………………………………………………………...25

• **Figure S10** Subgroup analyses of overall survival between the non-severe BMDD and severe BMDD groups. Hazard ratios were derived from univariate cox model for each subgroup. Dashed line indicates Hazard ratio of 1. BMDD, bone mineral density decrease; CI, confidence interval. ^*^Non-small cell lung cancer other than squamous cell carcinoma and adenocarcinoma…….…26

• **Figure S11** Heat map of correlation coefficients between the individual parameters. A, albumin; S, clinical stages; E, ECOG status; Co, corticosteroid use; P, PD-L1 expression; ^*^B, bone mineral density; ^#^B, bone mineral density decrease......................…27

• **Figure S12** Segmentation model training flow chart.…………………………………………………………………..………...28

**Table S1 ICI drugs used by patients in different BMD groups**

| Characteristics | Pathological BMD Group (n = 1391) | Physiological BMD Group (n = 705) | *P* Value | Severe BMDD Group (n = 822) | Non-severe BMDD Group (n = 1274) | *P* Value |
| --- | --- | --- | --- | --- | --- | --- |
| ICI drugs |  |  | .202 |  |  | .491 |
| Tislelizumab, n (%) | 432 (31.1%) | 188 (26.7%) |  | 231 (28.1%) | 389 (30.5%) |  |
| Sintilimab, n (%) | 376 (27.0%) | 199 (28.2%) |  | 232 (28.2%) | 343 (26.9%) |  |
| Camrelizumab, n (%) | 242 (17.4%) | 112 (15.9%) |  | 145 (17.6%) | 209 (16.4%) |  |
| Pembrolizumab, n (%) | 193 (13.9%) | 116 (16.5%) |  | 119 (14.5%) | 190 (14.9%) |  |
| Toripalimab, n (%) | 41 (2.9%) | 21 (3.0%) |  | 21 (2.6%) | 41 (3.2%) |  |
| Nivolumab, n (%) | 38 (2.7%) | 31 (4.4%) |  | 24 (2.9%) | 45 (3.5%) |  |
| Durvalumab, n (%) | 29 (2.1%) | 18 (2.6%) |  | 19 (2.3%) | 28 (2.2%) |  |
| Atezolizumab, n (%) | 14 (1.0%) | 9 (1.3%) |  | 13 (1.6%) | 10 (0.8%) |  |
| ^*^Other, n (%) | 26 (1.9%) | 11 (1.6%) |  | 18 (2.2%) | 19 (1.5%) |  |

BMD, bone mineral density; BMDD, bone mineral density decrease; ICI, immune checkpoint inhibitor.

^*^ICIs other than the above drugs.

**Table S2 DSC of the segmentation results of muscle, fat, and spine areas in the training and test sets**

| Segmentation Area | Training set DSC (95% CI) | Test set DSC (95% CI) |
| --- | --- | --- |
| Muscle | 0.931 (0.918, 0.942) | 0.910 (0.898, 0.921) |
| Fat | 0.936 (0.925, 0.948) | 0.914 (0.902, 0.928) |
| Spine | 0.954 (0.942, 0.967) | 0.937 (0.925, 0.950) |

CI, confidence interval.

**Table S3 Tumor Response** **in different BMD groups**

| Tumor response | Pathological BMD (n=1391) | Physiological BMD (n=705) | *P* value | Severe BMDD  (n=822) | Non-severe BMDD (n=1274) | *P* value |
| --- | --- | --- | --- | --- | --- | --- |
| CR, n | 0 | 0 |  | 0 | 0 |  |
| PR, n | 465 | 239 |  | 279 | 425 |  |
| SD, n | 750 | 385 |  | 439 | 696 |  |
| PD, n | 176 | 81 |  | 104 | 153 |  |
| DCR (%) | 87.3% | 88.5% | 0.443 | 87.3% | 88.0% | 0.661 |
| ORR (%) | 33.4% | 33.9% | 0.829 | 33.9% | 33.4% | 0.783 |

BMDA, age-adjusted standard bone mineral density; BMDD, bone mineral density decrease; CR, complete response; PR, partial response; SD, stable disease; PD, progressive disease; DCR, disease control rate; ORR, objective response rate.

**Table S4 Survival rate in different BMD groups at different time periods**

| Time period | Pathological BMD (n=1391) | Physiological BMD (n=705) | *P* value | Severe BMDD  (n=822) | Non-severe BMDD (n=1274) | *P* value |
| --- | --- | --- | --- | --- | --- | --- |
| One-year survival rate (%) | 79.5 (77.3, 81.8) | 80.4 (77.3, 83.5) | 0.359 | 76.6 (73.7, 79.6) | 82.0 (79.8, 84.3) | 0.007 |
| Two-year survival rate (%) | 54.0 (50.9, 57.2) | 61.3 (57.3, 65.5) | 0.009 | 53.3 (49.6, 57.2) | 58.9 (55.7, 62.4) | 0.029 |
| Three-year survival rate (%) | 35.7 (32.3, 39.4) | 49.7 (45.2, 54.6) | <0.001 | 35.7 (31.8, 40.1) | 45.0 (41.3, 49.0) | 0.002 |
| Five-year survival rate (%) | 14.1 (9.2, 21.5) | 22.6 (15.3, 33.3) | 0.100 | 13.3 (8.3, 21.3) | 22.8 (17.3, 30.1) | 0.045 |

Data are medians with interquartile ranges in parentheses unless otherwise indicated. BMDA, age-adjusted standard bone mineral density; BMDD, bone mineral density decrease.

**Table S5** **Univariate and multivariate Cox proportional hazards analyses for PFS**

| Parameter | Univariate analysis | | Multivariate analysis | |
| --- | --- | --- | --- | --- |
|  | Hazard ratio (95% CI) | *P* Value | Hazard ratio (95% CI) | *P* Value |
| Groups (BMD) |  |  |  |  |
| Physiological BMD | Reference |  | Reference |  |
| Pathological BMD | 1.19 (1.06, 1.34) | .003 | 1.20 (1.06, 1.35) | .003 |
| Groups (BMDD) |  |  |  |  |
| Non-severe BMDD | Reference |  |  |  |
| Severe BMDD | 1.19 (1.07, 1.32) | .002 | 1.24 (1.11, 1.39) | < .001 |
| Gender |  |  |  |  |
| Male | Reference |  |  |  |
| Female | 1.00 (0.86, 1.15) | .972 |  |  |
| Age | 1.00 (0.99, 1.00) | .612 |  |  |
| Pathological types |  |  |  |  |
| Adenocarcinoma | Reference |  |  |  |
| Squamous cell carcinoma | 0.93 (0.83, 1.04) | .202 |  |  |
| Other^*^ | 1.07 (0.90, 1.29) | .444 |  |  |
| Stages |  |  |  |  |
| Stage III | Reference |  | Reference |  |
| Stage IV | 1.40 (1.24, 1.57) | < .001 | 1.27 (1.11, 1.45) | < .001 |
| BMI (kg/m^2^) | 1.00 (0.98, 1.02) | .997 |  |  |
| Diabetes |  |  |  |  |
| No | Reference |  |  |  |
| Yes | 1.09 (0.91, 1.30) | .377 |  |  |
| Hypertension |  |  |  |  |
| No | Reference |  |  |  |
| Yes | 0.98 (0.86, 1.11) | .719 |  |  |
| Smoking |  |  |  |  |
| No | Reference |  |  |  |
| Yes | 0.96 (0.86, 1.07) | .487 |  |  |
| Drinking |  |  |  |  |
| No | Reference |  |  |  |
| Yes | 0.92 (0.80, 1.04) | .180 |  |  |
| Hyperlipidemia |  |  |  |  |
| No | Reference |  |  |  |
| Yes | 0.95 (0.83, 1.09) | .468 |  |  |
| Total bilirubin (μmol/L) | 1.00 (0.99, 1.01) | .689 |  |  |
| Albumin (g/L) | 0.97 (0.96, 0.98) | < .001 | 0.98 (0.97, 0.99) | < .001 |
| Calcium (mmol/L) | 0.94 (0.66, 1.35) | .751 |  |  |
| NLR | 1.01 (1.00, 1.02) | .051 | 1.00 (0.99, 1.01) | .799 |
| PLR | 1.00 (1.00, 1.00) | < .001 | 1.00 (1.00, 1.00) | .038 |
| PD-L1 expression |  |  |  |  |
| TPS ≥ 1% | Reference |  |  |  |
| TPS < 1% | 1.13 (0.94, 1.36) | .192 |  |  |
| ECOG status |  |  |  |  |
| 0 | Reference |  |  |  |

**Table S5** **Univariate and multivariate Cox proportional hazards analyses for PFS (continued)**

| Parameter | Univariate analysis | | Multivariate analysis | |
| --- | --- | --- | --- | --- |
|  | Hazard ratio (95% CI) | *P* Value | Hazard ratio (95% CI) | *P* Value |
| ≥1 | 1.45 (1.29, 1.63) | < .001 | 1.38 (1.22, 1.56) | < .001 |
| Bone metastasis |  |  |  |  |
| No | Reference |  | Reference |  |
| Yes | 1.44 (1.27, 1.63) | < .001 | 1.30 (1.14, 1.49) | < .001 |
| Brain metastases |  |  |  |  |
| No | Reference |  | Reference |  |
| Yes | 1.19 (1.02, 1.39) | .024 | 1.04 (0.89, 1.23) | .602 |
| Corticosteroid use |  |  |  |  |
| No | Reference |  |  |  |
| Yes | 1.16 (1.04, 1.29) | .008 | 1.13 (1.01, 1.27) | .034 |
| Immunotherapy regimen |  |  |  |  |
| ICI | Reference |  | Reference |  |
| ICI with chemotherapy | 0.87 (0.75, 1.02) | .086 | 0.87 (0.74, 1.02) | .095 |

All hazard ratios are shown for one-unit increments for each variable unless otherwise indicated. PFS, progression-free survival; CI, confidence interval; BMD, bone mineral density; BMDD, bone mineral density decrease; BMI, body mass index; NLR, Neutrophil to lymphocyte ratio; PLR, Platelet count to lymphocyte ratio; PD-L1, programmed cell death-ligand 1; TPS, tumor proportion score; ECOG, eastern cooperative oncology group; ICI, immune checkpoint inhibitor.

^*^Non-small cell lung cancer other than squamous cell carcinoma and adenocarcinoma.

**Table S6** **Univariate and multivariate Cox proportional hazards analyses for PFS with EGFR, ALK, KRAS and MET**

| Parameter | Univariate analysis | | Multivariate analysis^*^ | |
| --- | --- | --- | --- | --- |
|  | Hazard ratio (95% CI) | *P* Value | Hazard ratio (95% CI) | *P* Value |
| EGFR |  |  |  |  |
| - | Reference |  |  |  |
| + | 1.01 (0.68, 1.51) | .955 |  |  |
| ALK |  |  |  |  |
| - | Reference |  |  |  |
| + | 0.88 (0.39, 1.99) | .750 |  |  |
| KRAS |  |  |  |  |
| - | Reference |  | Reference |  |
| + | 0.50 (0.28, 0.89) | .019 | 0.55 (0.30, 1.00) | .052 |
| MET |  |  |  |  |
| - | Reference |  | Reference |  |
| + | 0.59 (0.31, 1.12) | .106 | 0.65 (0.34, 1.26) | .202 |

All hazard ratios are shown for one-unit increments for each variable unless otherwise indicated. PFS, progression-free survival; CI, confidence interval; BMD, bone mineral density; BMDD, bone mineral density decrease; PLR, Platelet count to lymphocyte ratio; ECOG, eastern cooperative oncology group.

^*^Multivariate analysis is adjusted for BMD, BMDD, stages, albumin, PLR, ECOG status, bone metastasis and corticosteroid use.

**Table S7** **Univariate and multivariate Cox proportional hazards analyses for OS with EGFR, ALK, KRAS and MET**

| Parameter | Univariate analysis | | Multivariate analysis^*^ | |
| --- | --- | --- | --- | --- |
|  | Hazard ratio (95% CI) | *P* Value | Hazard ratio (95% CI) | *P* Value |
| EGFR |  |  |  |  |
| - | Reference |  |  |  |
| + | 1.06 (0.63, 1.79) | .823 |  |  |
| ALK |  |  |  |  |
| - | Reference |  |  |  |
| + | 0.99 (0.36, 2.70) | .984 |  |  |
| KRAS |  |  |  |  |
| - | Reference |  |  |  |
| + | 0.90 (0.45, 1.80) | .771 |  |  |
| MET |  |  |  |  |
| - | Reference |  |  |  |
| + | 1.18 (0.59, 2.36) | .650 |  |  |

All hazard ratios are shown for one-unit increments for each variable unless otherwise indicated. OS, overall survival; CI, confidence interval; BMD, bone mineral density; BMDD, bone mineral density decrease; PD-L1, programmed cell death-ligand 1; ECOG, eastern cooperative oncology group.

^*^Multivariate analysis is adjusted for BMD, BMDD, stages, albumin, PD-L1 expression, ECOG status and corticosteroid use.

**Table S8 Correlation coefficient between the individual parameters**

|  | Albumin | Stages | PD-L1 expression | ECOG status | Corticosteroid use | Group (BMD) | Group (BMDD) |
| --- | --- | --- | --- | --- | --- | --- | --- |
| Albumin |  | 0.574 | 0.350 | 0.236 | 0.566 | 0.349 | 0.338 |
| Stages | rpb=0.013 |  | 0.286 | 0.050 | 0.006 | 0.606 | 0.496 |
| PD-L1 expression | rpb=-0.034 | φ=-0.039 |  | <0.001 | 0.153 | 0.114 | 0.577 |
| ECOG status | rpb=-0.026 | φ=0.043 | φ=0.201 |  | <0.001 | 0.504 | 0.758 |
| Corticosteroid use | rpb=-0.013 | φ=0.060 | φ=0.052 | φ=0.117 |  | 0.426 | 0.842 |
| Group (BMD) | rpb=0.021 | φ=-0.011 | φ=0.057 | φ=-0.015 | φ=-0.017 |  | <0.001 |
| Group (BMDD) | rpb=-0.021 | φ=0.015 | φ=0.020 | φ=-0.007 | φ=0.004 | φ=-0.094 |  |

The lower half of the table is the correlation coefficient, and the upper half of the table is the statistical p value. The result of Point-Biserial Correlation is expressed as rpb, and the result of Phi correlation is expressed as φ. BMD, bone mineral density; BMDD, bone mineral density decrease.

**Table S9** **CT scanners and parameters of the five institutions**

| Parameter | WHUH cohort | AHNU cohort | FJ900H cohort | GYFYY cohort | SZPH cohort |
| --- | --- | --- | --- | --- | --- |
| CT scanners | Siemens, Philips, Toshiba, GE | Siemens, Philips, GE | Siemens, Philips | Siemens, GE, Neosoft | Philips |
| Slice thickness | 0.625-5 mm | 1.25-2 mm | 1.25-2 mm | 1-5 mm | 1.5mm |
| Slice interval | 1-1.5 mm | 1-1.5 mm | 1-1.5 mm | 0.8-1 mm | 1 mm |
| Detector rows | 64, 128, 256-section | 64, 128, 256-section | 64, 128-section | 32, 64, 128, 256-section | 128-section |
| Tube current | Auto mA | 150~190 mA | 148~252 mA | 150-361mA | Auto mA |
| Tube voltage | 70-140 kV | 120 kV | 120-140 kV | 60-140 kV | 120 kV |
| Data matrix | 512 × 512 | 512 × 512 | 512 × 512 | 512 × 512 | 512 × 512 |
| Helical pitch | 0.03-1.5 | 0.15-1.5 | 0.16-1 | 0.8-1.2 | 1.383 |

WHUH, Wuhan Union Hospital; AHNU, Affiliated Hospital of Nantong University; FJ900H, Fujian 900th Hospital of Joint Logistics Support Force; GYFYY, The First Affiliated Hospital of Guangzhou Medical University; SZPH, Shenzhen People's Hospital.

**Table S10 Segmentation model training parameters**

| parameters |  |
| --- | --- |
| Batchsize | 1 |
| Learning Rate | StepLR |
| Epoch | 5 |
| Optimizer | SGD, Adam |
| Loss Function | CE Loss, Focal Loss |


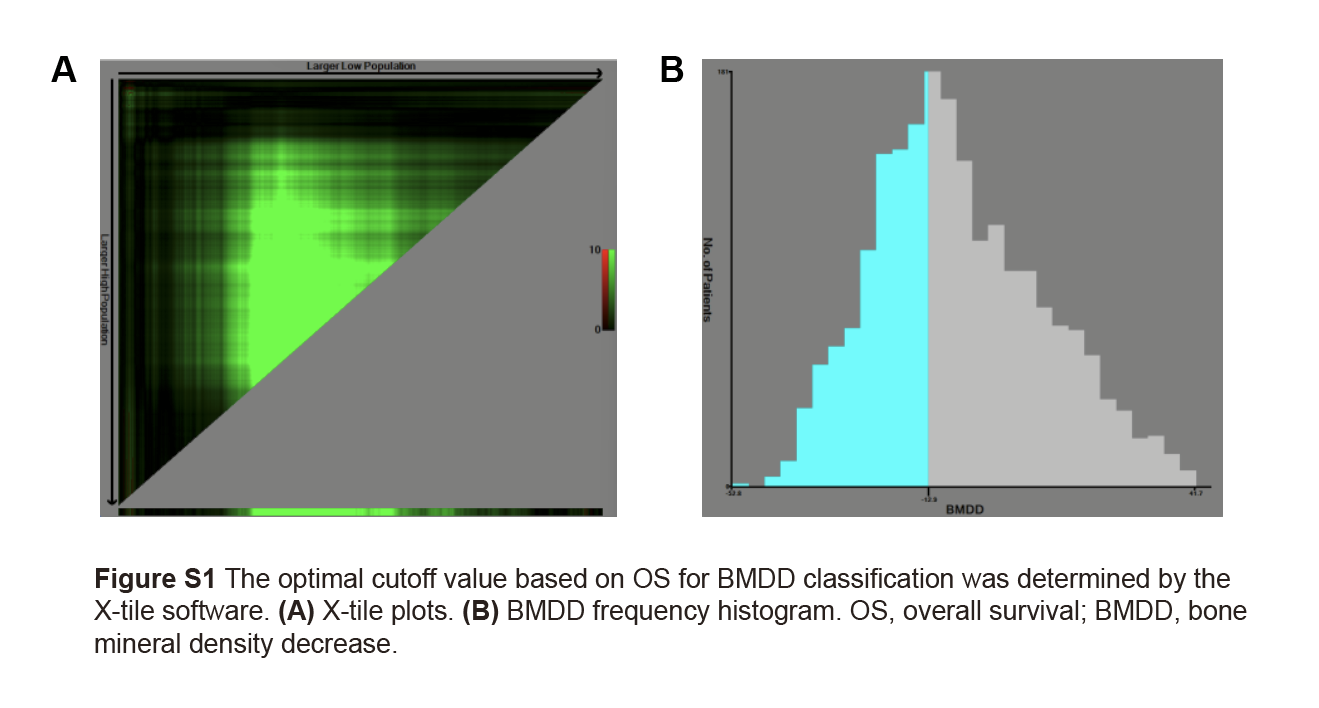


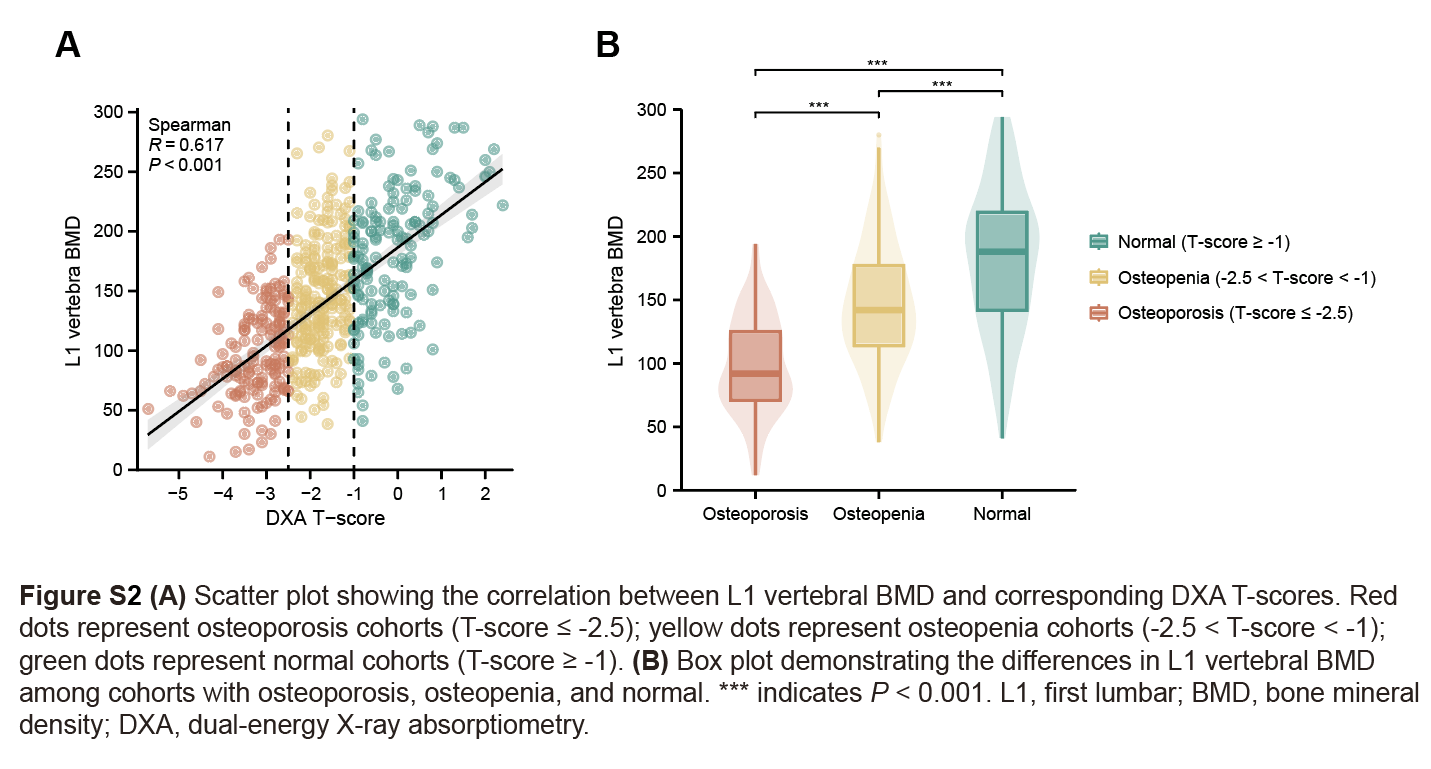


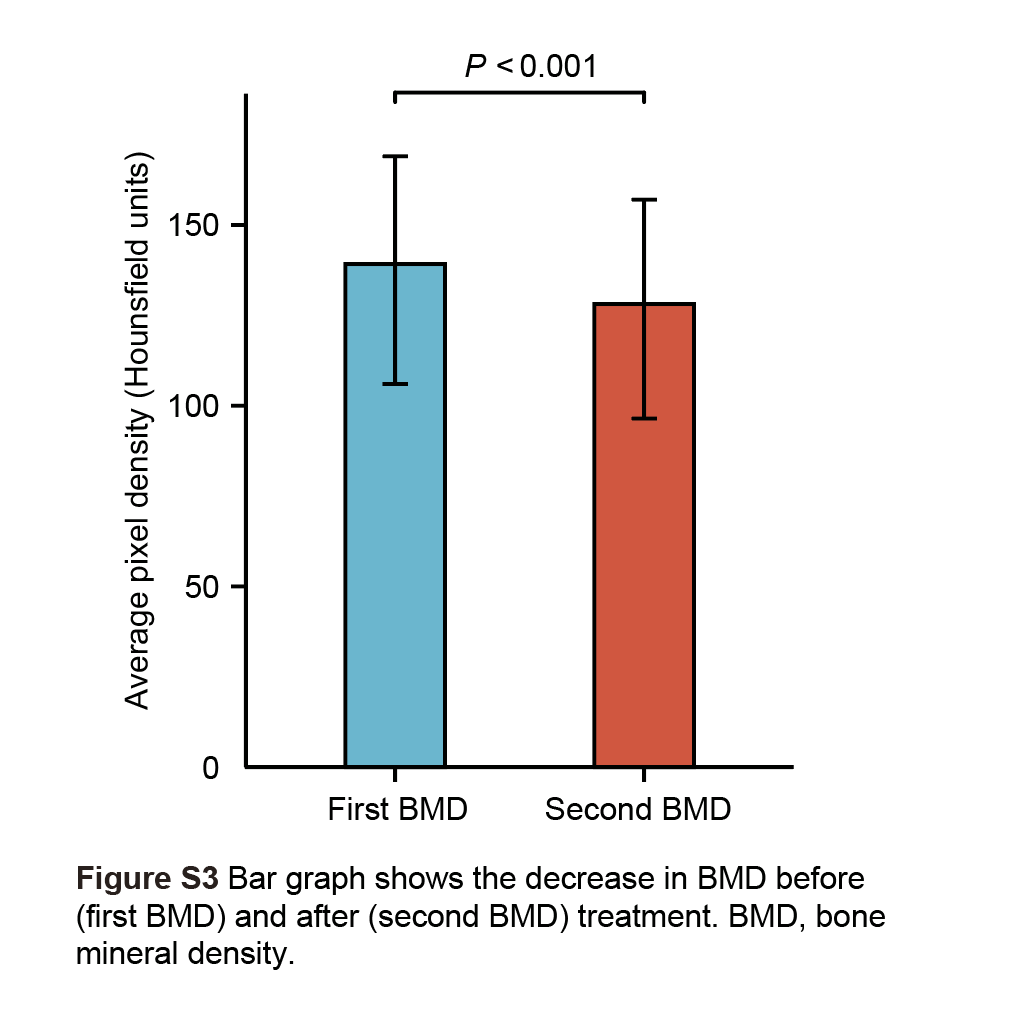


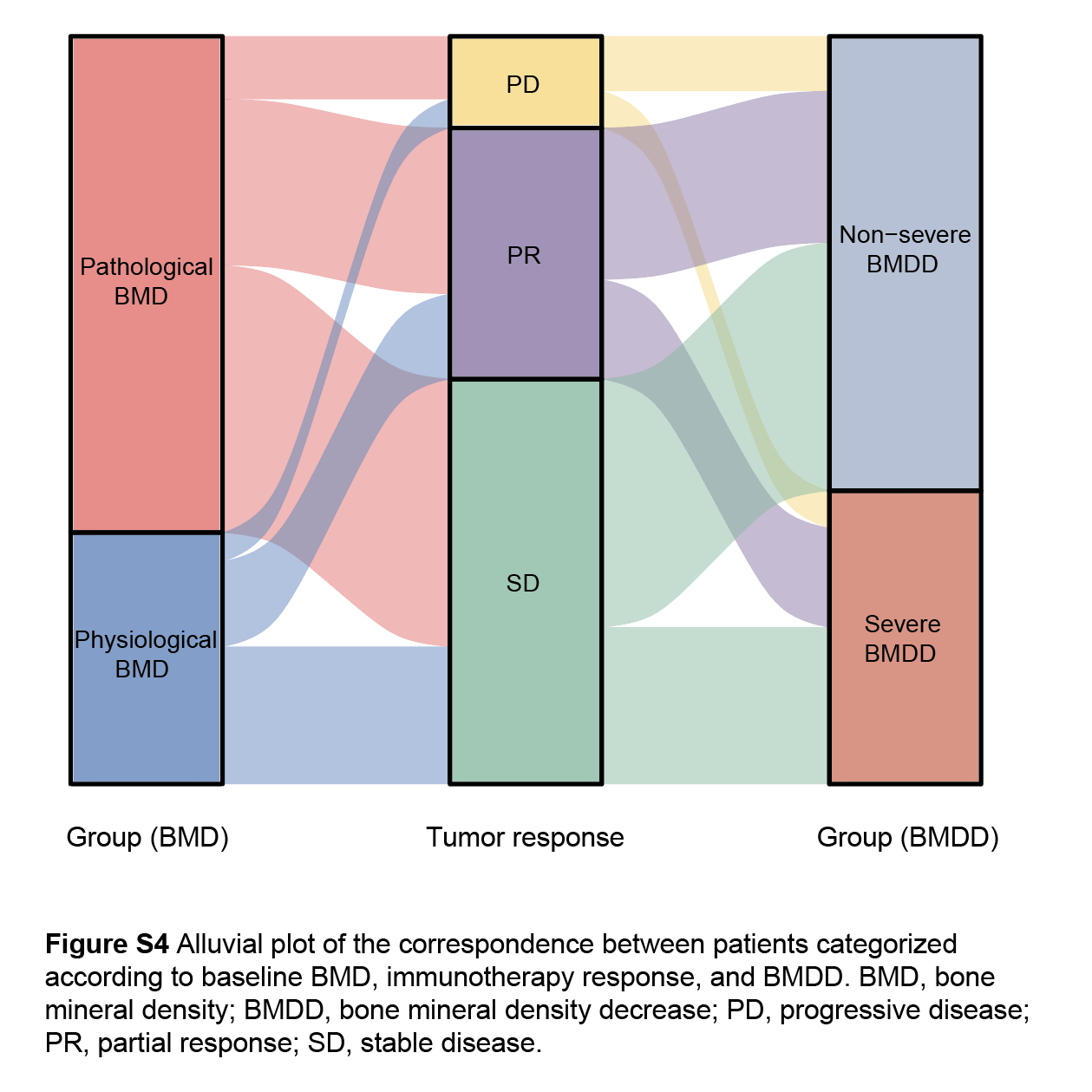


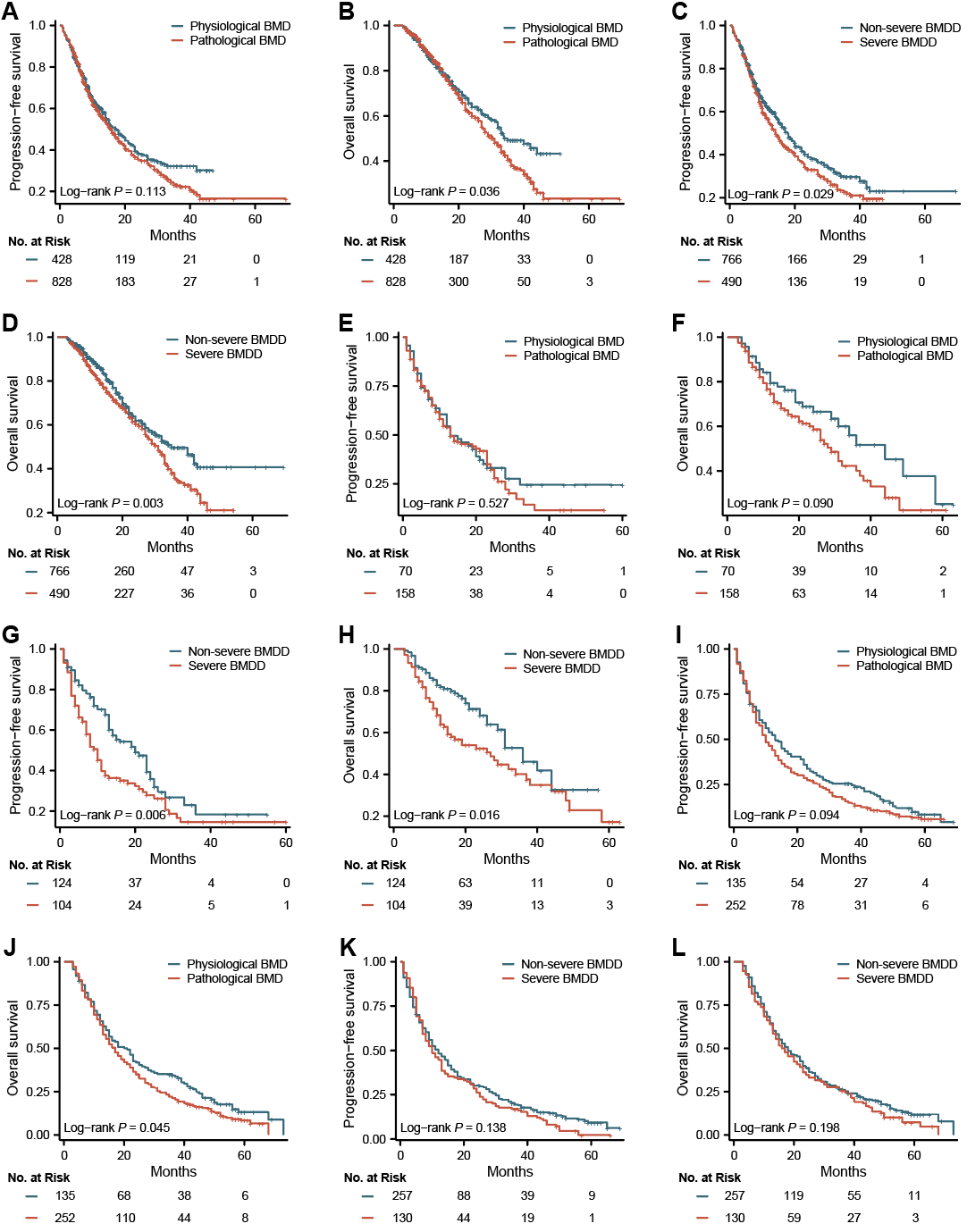


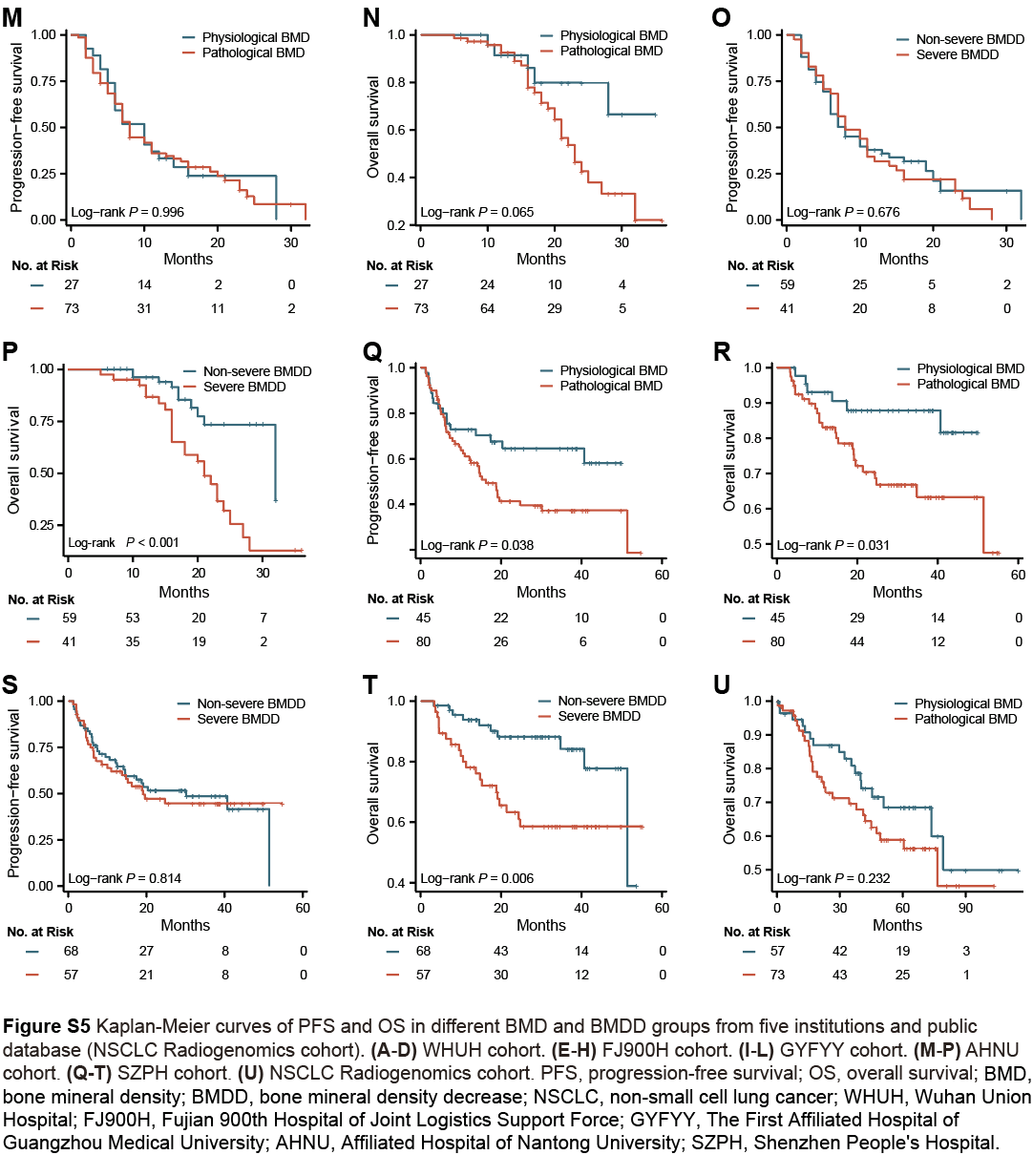


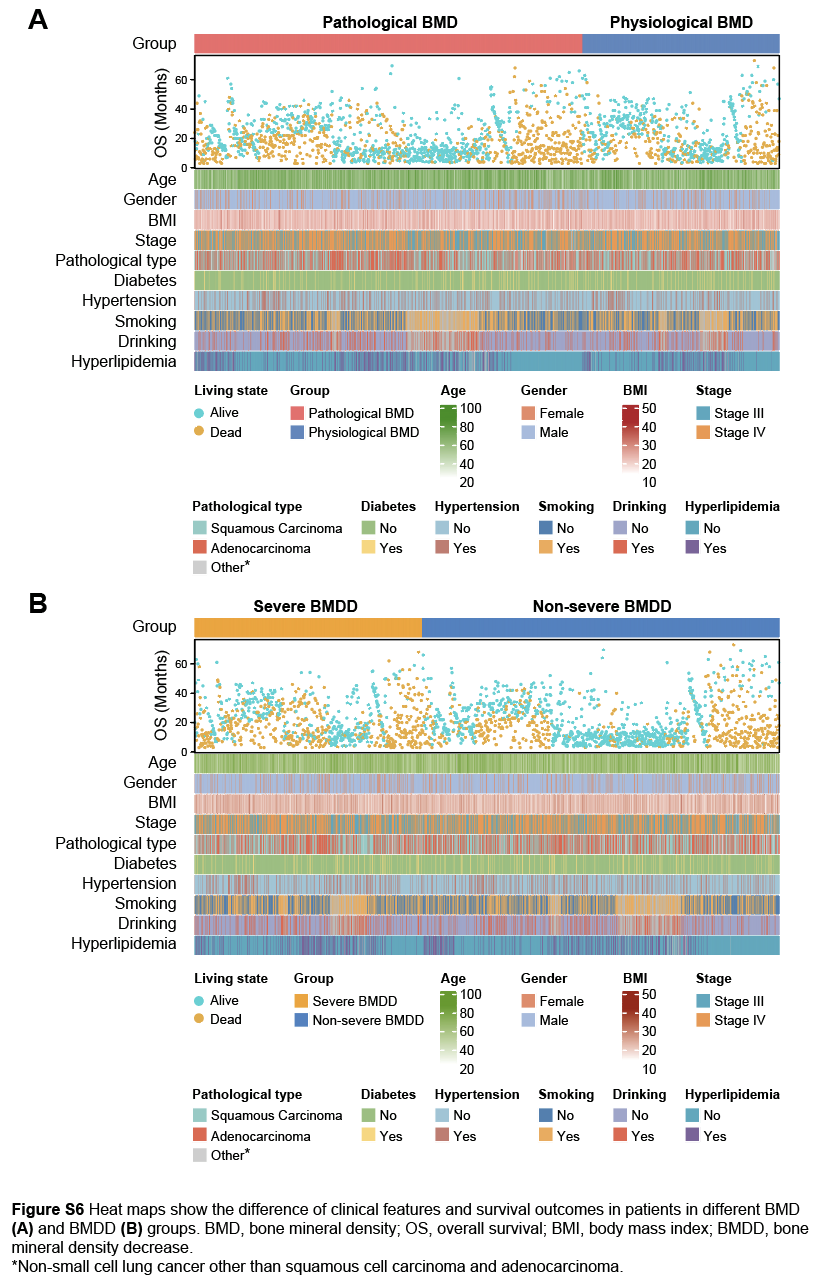


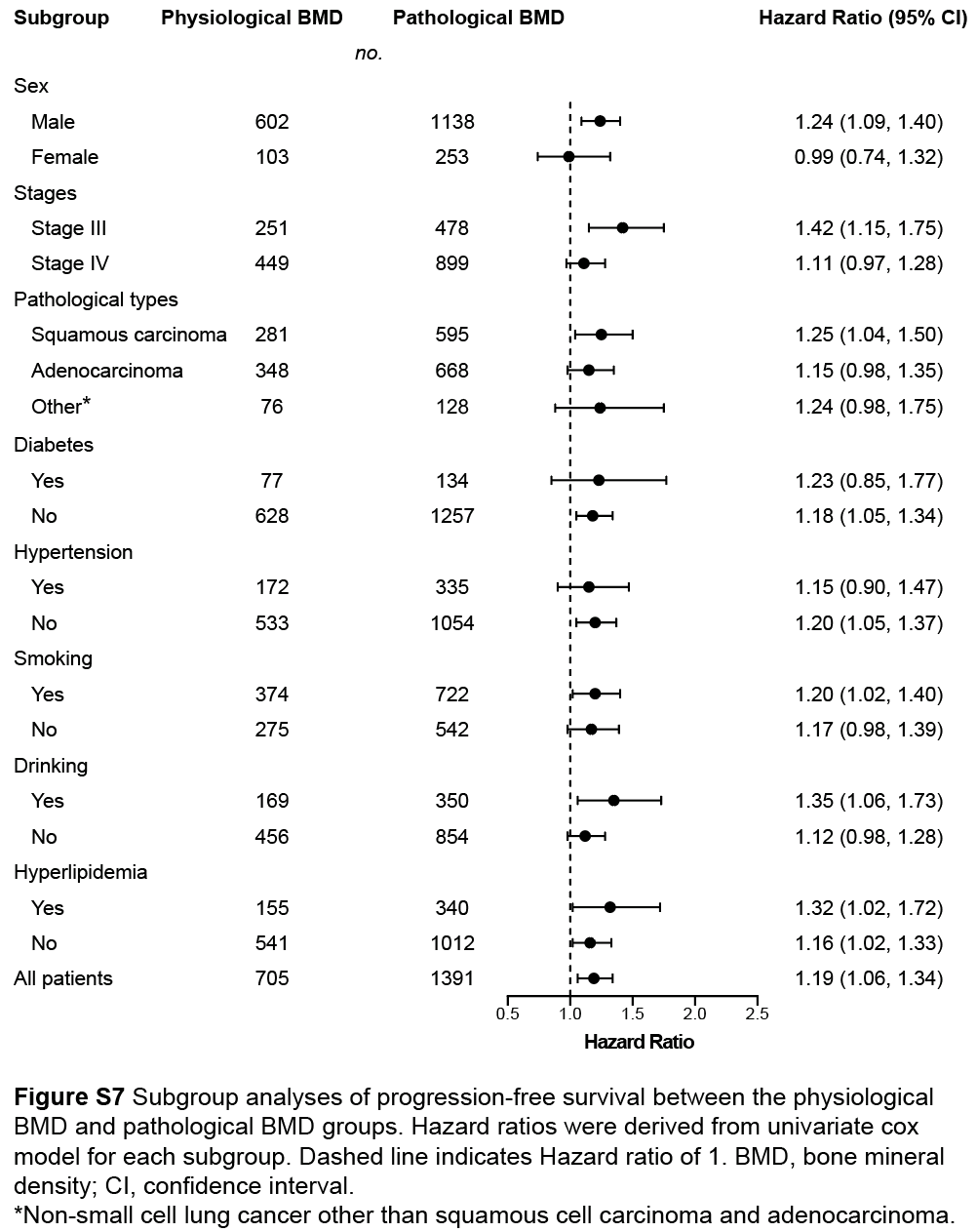


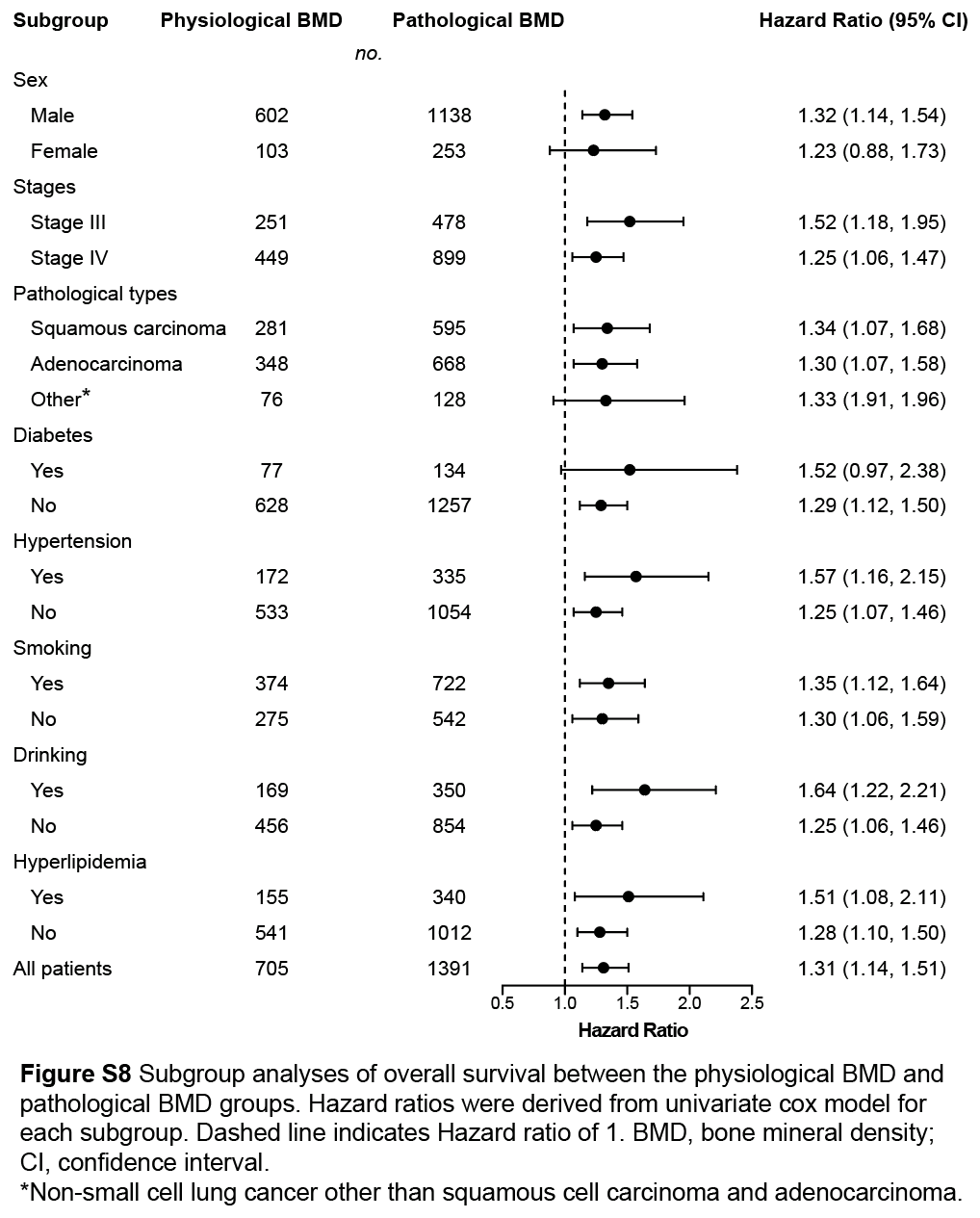


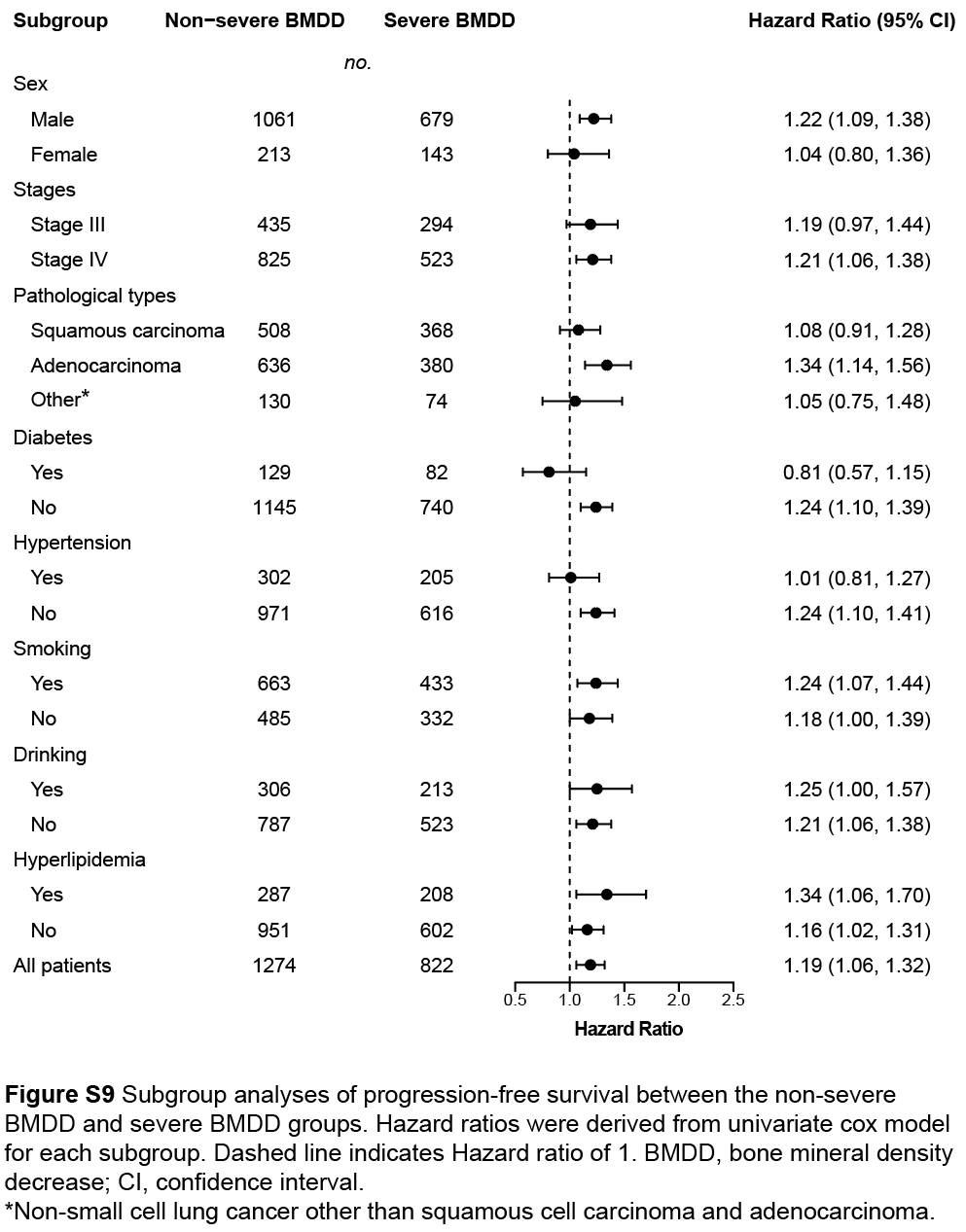


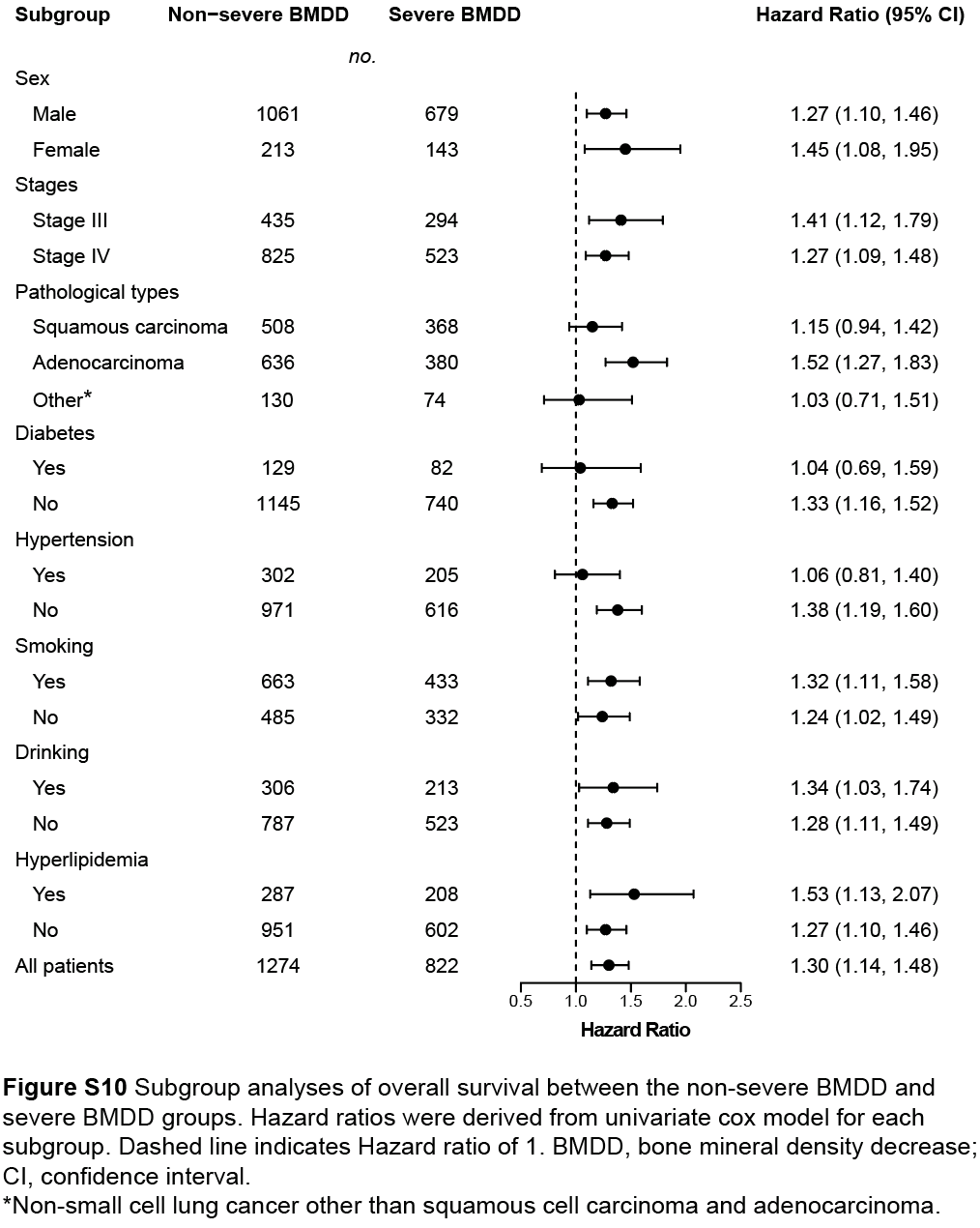


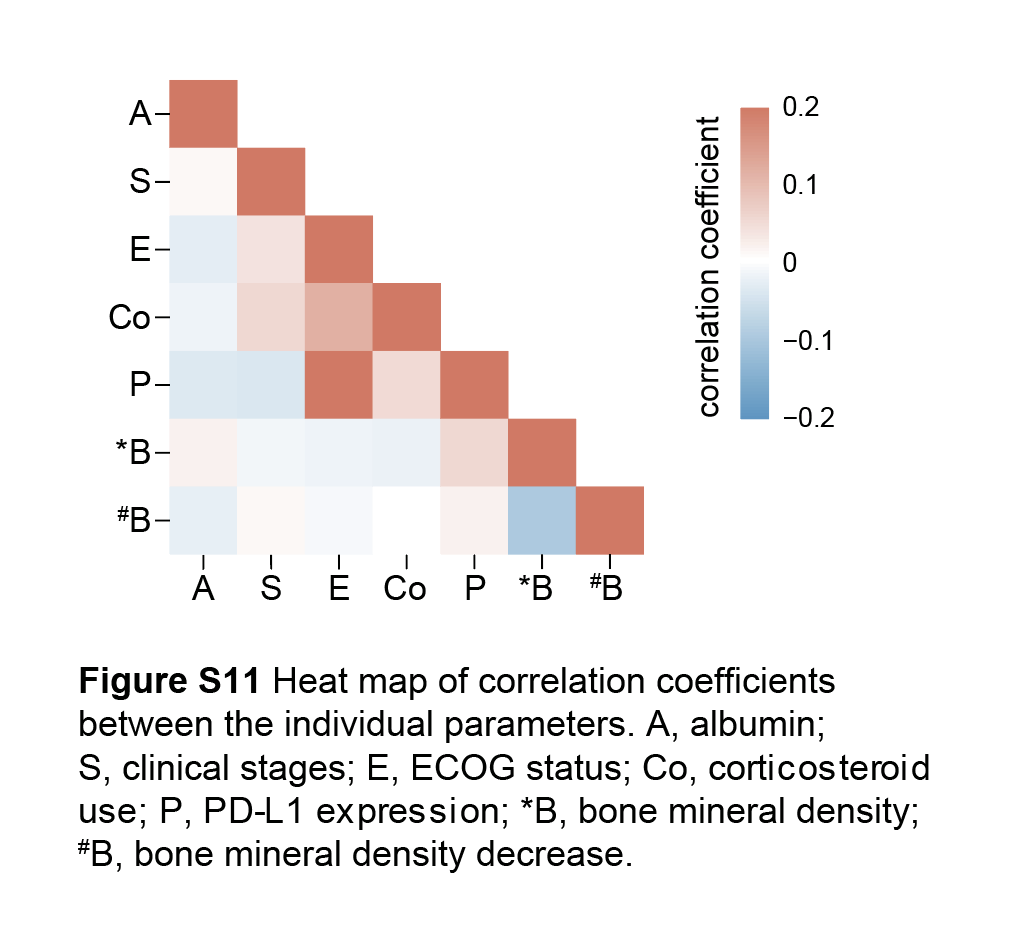

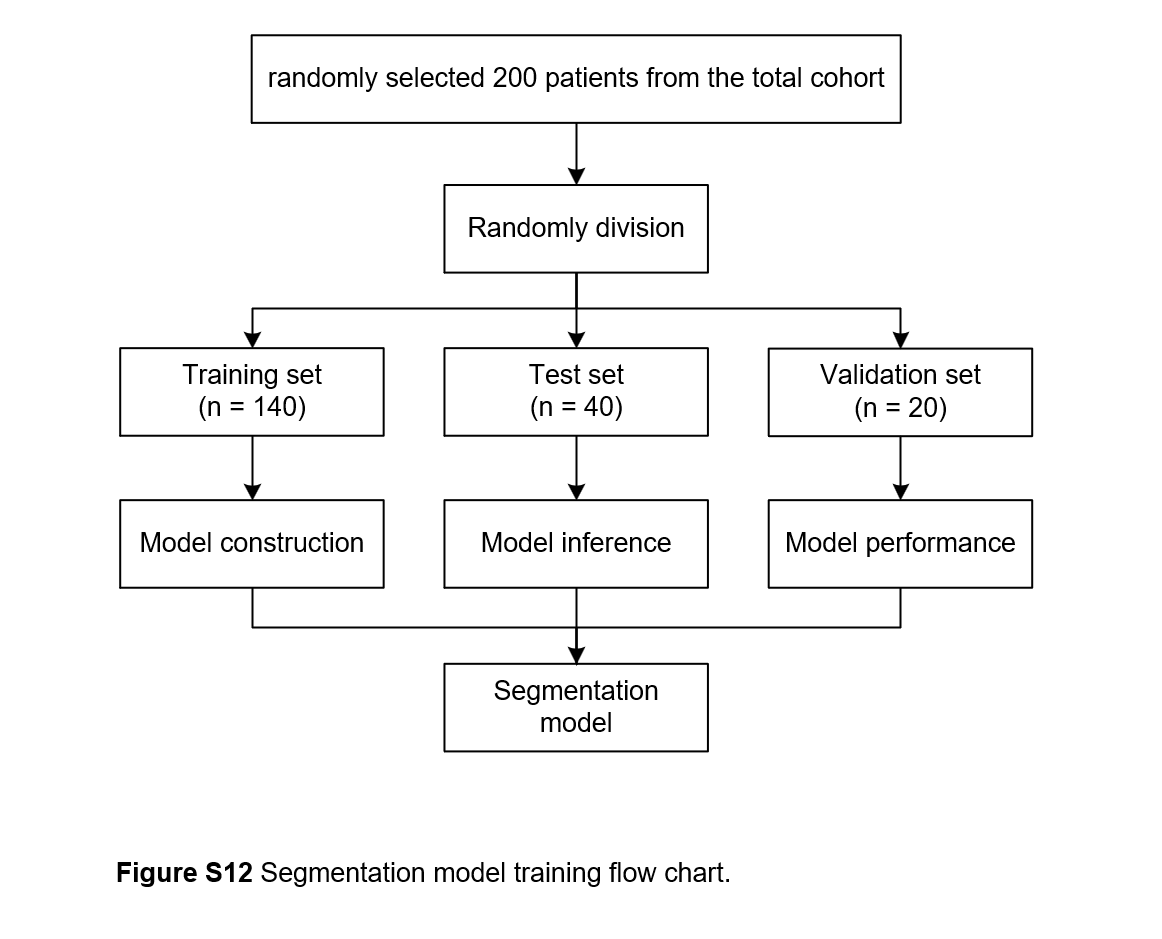

Supplement: Supplementary file 1 — Table S1: ICI drugs used by patients in different BMD groups.Table S2: DSC of the segmentation results of muscle, fat, and spine areas in the training and test sets.Table S3: Tumor Response in different BMD groups.Table S4: Survival rate in different BMD groups at different time periods.Table S5: Univariate and multivariate Cox proportional hazards analyses for PFS.Table S6: Univariate and multivariate Cox proportional hazards analyses for PFS with EGFR, ALK, KRAS and MET.Table S7: Univariate and multivariate Cox proportional hazards analyses for OS with EGFR, ALK, KRAS and MET.Table S8: Correlation coefficient between the individual parameters.Table S9: CT scanners and parameters of the five institutions.Table S10: Segmentation model training parameters.Figure S1: The optimal cutoff value based on OS for BMDD classification was determined by the X‐tile software. (A) X‐tile plots. (B) BMDD frequency histogram. OS, overall survival; BMDD, bone mineral density decrease.Figure S2 (A): Scatter plot showing the correlation between L1 vertebral BMD and corresponding DXA T‐scores. Red dots represent osteoporosis cohorts (T‐score ≤ ‐2.5); yellow dots represent osteopenia cohorts (‐2.5 < T‐score < ‐1); green dots represent normal cohorts (T‐score ≥ ‐1). (B) Box plot demonstrating the differences in L1 vertebral BMD among cohorts with osteoporosis, osteopenia, and normal. *** indicates p < 0.001. L1, first lumbar; BMD, bone mineral density; DXA, dual‐energy X‐ray absorptiometry.Figure S3: Bar graph shows the decrease in BMD before (first BMD) and after (second BMD) treatment. BMD, bone mineral density.Figure S4: Alluvial plot of the correspondence between patients categorized according to baseline BMD, immunotherapy response, and BMDD. BMD, bone mineral density; BMDD, bone mineral density decrease; PD, progressive disease; PR, partial response; SD, stable disease.Figure S5: Kaplan‐Meier curves of PFS and OS in different BMD and BMDD groups from five institutions and public da [file MCO2-6-e70398-s001.docx]
